# Utility of Three-Coordinate Silver Complexes Toward the Formation of Iodonium Ions

**DOI:** 10.1021/acs.inorgchem.1c00409

**Published:** 2021-03-25

**Authors:** Jas S. Ward, Antonio Frontera, Kari Rissanen

**Affiliations:** † Department of Chemistry, University of Jyvaskyla, Jyväskylä 40014, Finland; ‡ Department of Chemistry, Universitat de les Illes Balears, Crts de Valldemossa km 7.6, 07122 Palma de Mallorca, Baleares, Spain

## Abstract

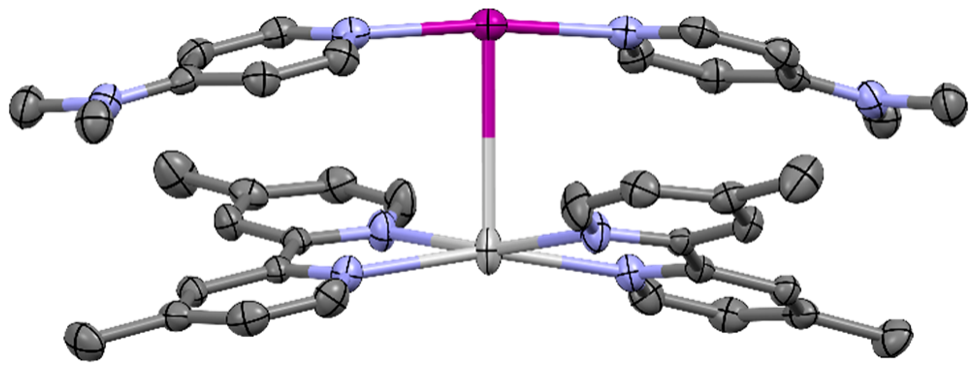

The work herein describes the synthesis of five three-coordinate silver(I) complexes comprising a bidentate ligand L1, either bpy (2,2′-bipyridyl) or bpyMe_2_ (4,4′-dimethyl-2,2′-dipyridyl), and a monodentate ligand L2, either mtz (1-methyl-1H-1,2,3-triazole), 4-Etpy (4-ethylpyridine), or 4-DMAP (*N*,*N*-dimethylpyridin-4-amine). Upon reaction of the three-coordinate silver(I) complexes with 0.5 equiv of I_2_, the reactions quantitatively produce a 1:1 pair of complexes of a four-coordinate silver(I) complex [Ag(L1)_2_]PF_6_ and a two-coordinate iodonium complex [I(L2)_2_]PF_6_. The combination of [Ag(bpyMe_2_)_2_]PF_6_ and [I(4-DMAP)_2_]PF_6_ gave rise to an I^+^···Ag^+^ interaction where the I^+^ acts as a nucleophile, only the second example of which, that was observed in both the solution (NMR) and solid (X-ray) states.

## Abstract 2

The second example of an iodonium ion acting as a nucleophile, in both the solution and solid states, has been synthesized from a three-coordinate silver(I) complex via partial cation exchange.

## Introduction

The pursuit of new halonium ion motifs has been ongoing since their popularization in the 1990s,^1−4^ three decades after first being described in the literature,^5,6^ which was fueled by Barluenga using *his* reagent [I(pyridine)_2_]BF_4_ (Barluenga’s reagent) to demonstrate their great utility toward a myriad of organic transformations such as the electrophilic iodonation of unactivated arenes, the promotion of C–C and C–X bond formation, and the selective direct iodonation of peptides.^7−9^ However, while the reactivity of halonium ions is well explored territory, the properties of these species in themselves are less well studied, undoubtedly due to difficulties that arise from the aforementioned reactivity.

The formation of X^+^ halonium ions (X = Br, I) via a cation exchange process is well established,^4,10−13^ where the respective two-coordinate silver(I) complex is first synthesized and then reacted with elemental halogens X_2_ (X = Br, I) to yield the desired two-coordinate halonium complex through loss of AgX. This process can be extended to chlorine; however, the reactivity increases (and stability decreases) in the order I > Br ≫ Cl, which is reflected in the literature with very few examples of *chloronium* (Cl^+^) ions existing.^2,14,15^

**Figure 1:**
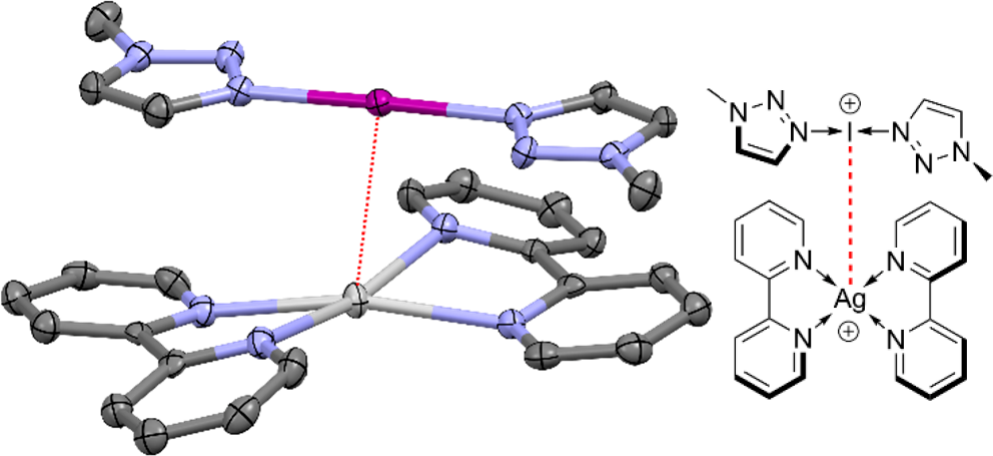
Single-crystal X-ray structure of the first I^+^—Ag^+^ interaction (3.4608(3) Å; indicated with a red dotted line) of an iodonium ion acting as a nucleophile (thermal ellipsoids at 50% probability; PF_6_ anions and hydrogen atoms omitted for clarity). Color key: purple = iodine, light gray = silver, blue = nitrogen, dark gray = carbon.

While three-coordinate silver(I) complexes are not as common as their two- and four-coordinate counterparts, they are still well accounted for in the literature.^16−19^ However, their use as precursors toward the synthesis of halonium ions via cation exchange was only recently reported,^20^ opening up the possibility of a new pool of potential silver(I) precursors that could be used to synthesize desirable halonium complexes. The derivatives of this first example of a three-coordinate silver(I) complex successfully reacting to a combination of a halonium ion and a silver(I) complex, by what could be described as a *partial* cation exchange, demonstrated a highly interesting and previously unknown interaction in which the I^+^ was acting as a nucleophile toward the Ag^+^ (I^+^···Ag^+^ = 3.4608(3) Å; Figure 1). Utilizing the same strategy of performing a partial cation exchange on a three-coordinate silver(I) complex, herein, the second example of this “nucleophilic” I^+^ interaction is reported between a pair of complexes synthesized directly from a three-coordinate silver(I) precursor. The nature of the I^+^···Ag^+^ has been analyzed using DFT calculations (M06-2X/def2-QZVP) combined with the quantum theory of “atoms-in-molecules” (QTAIM), the noncovalent interaction plot (NCIPlot) index, and the natural bond orbital (NBO) analyses.

## Results and Discussion

The first examples of heteroleptic halonium complexes highlighted that halonium ions can,^21−24^ if suitable ligands are present, undergo ligand scrambling in solution.^25^ It has also been reported that silver(I) complexes bearing bidentate ligands such as [Ag(bpy)_2_]PF_6_ (bpy = 2,2′-bipyridyl) are resistant to such processes and remained steadfastly coordinated to the Ag^+^ in solution when either I_2_ or iodonium ions were added.^20^ Therefore, the pursuit of the nucleophilic I^+^ interaction would be facilitated by testing *bis*(bidentate)silver(I) complexes as potential Lewis acid acceptors. The partial cation exchange process of three-coordinate silver(I) complexes adeptly yields the stoic *bis*(bidentate)silver(I) complexes concomitantly with the iodonium ion in the desired 1:1 stoichiometry, making them ideal to generate pairs of complexes in search for more instances of nucleophilic halonium interactions. This strategy bypasses the need to separately synthesize and, more challengingly, isolate and quantify the often highly reactive halonium species. Instead generating the reactive halonium ion *in situ* only when it is required. The combination of a monodentate and a bidentate ligand also brings the problem of ligand scrambling to heel, whereupon it works with the strategy toward the desired outcome, rather than against it.

**Scheme 1:**
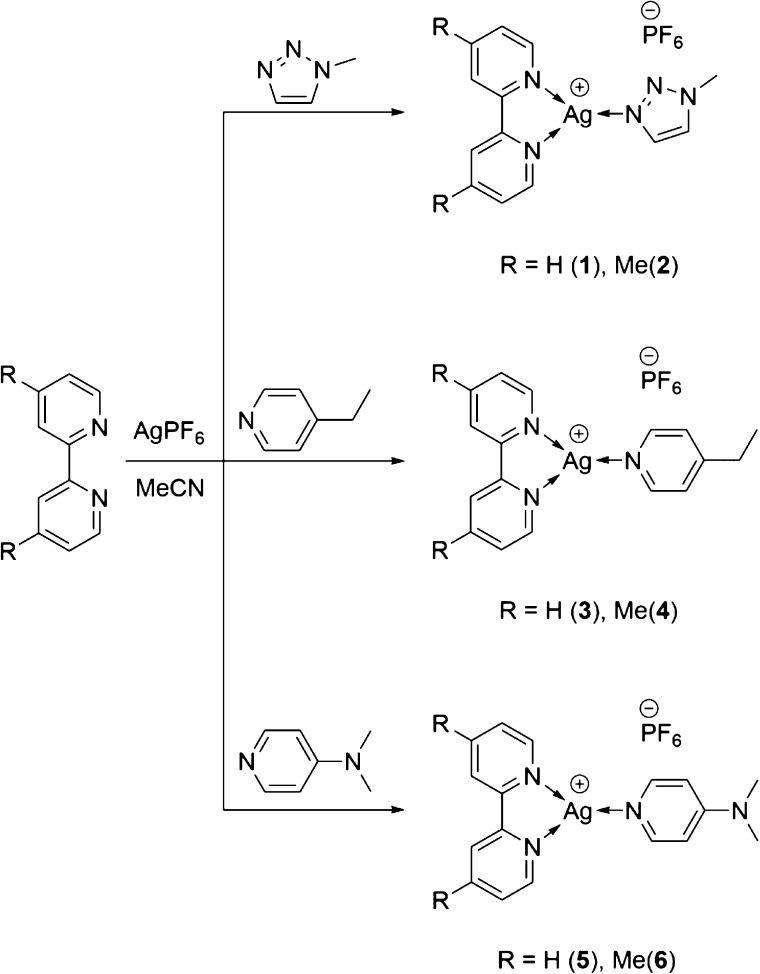
General Synthetic Procedure Used to Synthesize the Three-Coordinate Silver(I) Complexes **1**–**6**

The new three-coordinate silver(I) complexes **2**–**6** were synthesized straightforwardly in good yields and show no degradation over time as solids (Scheme 1), though some care must be taken due to their mild light sensitivity while they are in solution. The ^1^H NMR spectra of complexes **2**–**6** were as expected and warrant no further comment. The ^15^N NMR shifts were determined by ^1^H–^15^N HMBC NMR experiments, and the bpy nitrogen atoms in complexes **1** (−111.5 ppm), **3** (−110.1 ppm), and **5** (−108.7 ppm) were found not to deviate more than 5 ppm from the bpy resonances of [Ag(bpy)_2_]PF_6_ (*cf*. – 106.5 ppm), with the largest deviation found to be for the mtz (1-methyl-1H-1,2,3-triazole) derivative **1**. A similar trend of the resonances for the bpyMe_2_ (4,4′-dimethyl-2,2′-dipyridyl) nitrogen atoms not deviating more than 5 ppm from those of [Ag(bpyMe_2_)_2_]PF_6_ (*cf*. – 114.4 ppm) was also observed for complexes **2** (−119.2 ppm), **4** (−117.9 ppm), and **6** (−116.5 ppm), once again with the largest deviation observed for the mtz derivative **2**.

**Figure 2:**
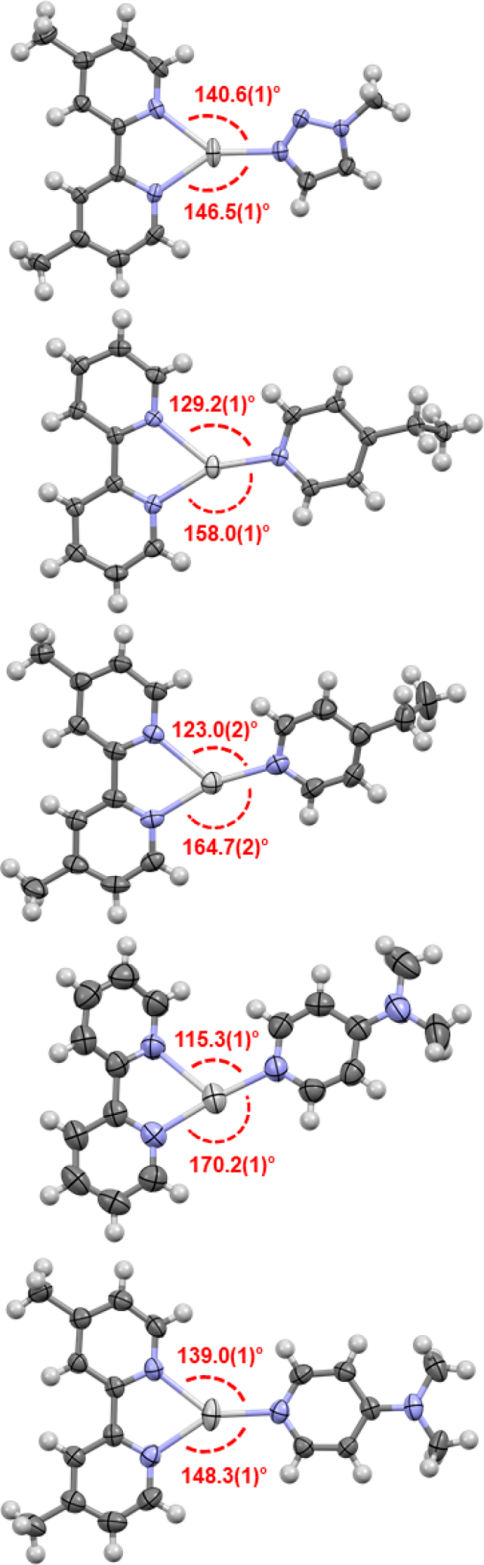
Single-crystal X-ray structures of complexes **2**–**6**, annotated with the N(monodentate)–Ag^+^–N(bidentate) angles in red (thermal ellipsoids at 50% probability; PF_6_ anions omitted for clarity). Color key: light gray = silver, blue = nitrogen, dark gray = carbon, white = hydrogen.

All three-coordinate geometries for complexes **2**–**6** were confirmed in the solid state by single-crystal X-ray diffraction (Figure 2). Complexes **2** and **6** were observed as discrete “monomeric” complexes, **3** and **5** were observed as argentophilic dimers with Ag^+^—Ag^+^ distances of 3.1664(5) and 3.231(3) Å, respectively, and **4** was observed as an argentophilic polymer (Ag^+^—Ag^+^ distances of 3.3314(7) and 3.3526(8) Å). The monodentate (mtz, 4-Etpy (4-ethylpyridine), 4-DMAP (*N*,*N*-dimethylpyridin-4-amine)), and bidentate (bpy, bpyMe_2_) ligands were found to be effectively coplanar in all complexes (**2**–**6**), with the exception of one ring of a bpy ligand in **5** (of one of the two crystallographically independent molecules present in the asymmetric unit cell of **5**) deviating significantly out of the plane by 33.7°. The coordination geometries were predominantly observed to be distorted trigonal planar for **2**, **3**, **6**, and one of the two crystallographically independent molecules of **4** and **5**, with N(monodentate)–Ag^+^–N(bidentate) angles in the range of 129.2(1)–158.0(1)°. However, one of the two crystallographically independent molecules of **4** and **5** did display significant deviation of this trigonal planar geometry with the example in **4** having N(monodentate)–Ag^+^–N(bidentate) angles of 123.0(2) and 164.7(2)° and the example in **5** having the more divergent angles of 115.3(1) and 170.2(1)° (it should be noted that in both of these instances, the AgN_3_ planes remain effectively planar, with the aforementioned deviations occurring within the plane). These two examples can be viewed as a distorted linear geometry between the monodentate ligand and one of the two coordinating pyridyl groups of the bidentate ligand, with the second pyridyl being only a pendant group that was only weakly coordinating to the Ag^+^, evidenced by their much longer Ag^+^–N bond lengths of 2.374(4) Å for **4** and 2.535(4) for **5** (*cf*. all other Ag^+^–N(bidentate) bonds that are within the range 2.249(4)–2.335(3) Å).

**Scheme 2:**
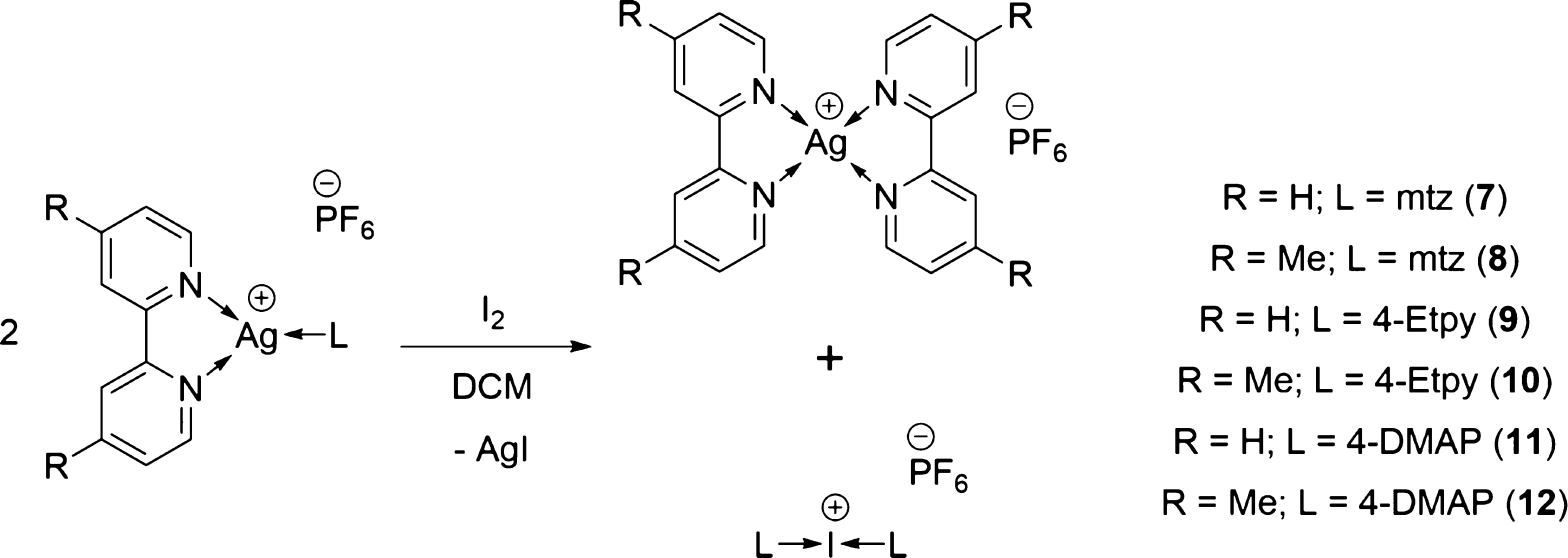
General Synthetic Procedure Used to Synthesize the Pairs of Complexes **7**–**12**

In the same manner as reported for the pair of complexes **7**, two equivalents of the three-coordinate silver(I) complexes (**2**–**6**) were reacted with 1 equiv of elemental iodine to generate the pairs of complexes **8**–**12** (Scheme 2).

**Figure 3:**
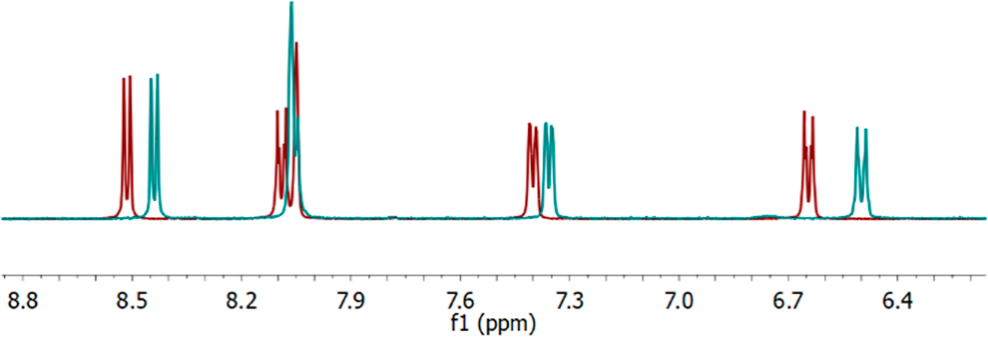
Superimposed ^1^H NMR spectra (between 6 and 9 ppm) of complex **6** (red) and the resulting pair of complexes **12** (cyan) that resulted after addition of 0.5 equiv of I_2_.

The partial cation exchanges proceeded rapidly upon addition of the I_2_, with immediate precipitation of AgI observed, and all reactions were found to have gone to completion within 5 min by ^1^H NMR spectroscopy. The ^1^H NMR spectra had observable shift changes of all resonances for the pairs of complexes (**8**–**12**) relative to their three-coordinate silver(I) precursors (**2**–**6**) with modest, but definitive, shifts (Figure 3) observed upon formation of the pairs of complexes, with maximum shifts changes (Δδ_H_) observed for the aromatic hydrogen atoms of 0.07 ppm (**4 → 10**), 0.08 ppm (**3 → 9**, **6 → 12**), 0.09 ppm (**2 → 8**), and 0.14 ppm (**5 → 11**). The identity of all the pairs of complexes (**8**–**12**) as such was readily determined by ^1^H NMR spectroscopy by comparison to the spectra of their respective individual pure I^+^ and Ag^+^ components, which had all been described previously in the literature with the exception of [Ag(bpyMe_2_)_2_]PF_6_ (**13**), that was subsequently synthesized and fully characterized for that explicit purpose. Only very minor differences, that could be considered within the error of the measurements and therefore negligible, were observed in the ^1^H NMR spectra when comparing the respective individual pure I^+^ and Ag^+^ components to the pairs of complexes (**8**–**12**), which was similarly observed in the effectively indistinguishable ^15^N NMR values for **8**, **9**, **10**, and **11** (Tables S1 and S2). However, as was reported for the pair of complexes **7** which displayed a −2.9 ppm shift of the bpy nitrogen atoms in [Ag(bpy)_2_]PF_6_ upon addition of 1 equiv of [I(mtz)_2_]PF_6_, a similar divergence was observed for the pair of complexes **12**. A ^15^N NMR chemical shift change of 2.3 ppm was observed between that of the individual complex **13** (−114.4 ppm) and the [Ag(bpyMe_2_)_2_]PF_6_ component of the pair of complexes **12** (−112.1 ppm), indicating the presence of an I^+^···Ag^+^ interaction in solution. It should be noted that no distinguishable ^15^N NMR chemical shift change was observed for the [I(4-DMAP)_2_]PF_6_ component of **12** (−216.0 ppm) when compared to the value of the individual spectrum of [I(4-DMAP)_2_]PF_6_ (−216.1 ppm), once again reminiscent of what was observed for the pair of complexes **7**.

The pair of complexes **7** was reported to form a nucleophilic iodonium interaction that was observed in both the solution and solid states. Extensive attempts were made to generate similarly interacting cocrystals of all pairs of complexes (**8**–**12**) generated in this study, varying conditions such as solvents, antisolvents, and the crystallization method used, though for **8**, **9**, and **11** only crystals of their respective pure Ag^+^ or I^+^ components were observed. A noninteracting cocrystal **10** was successfully generated, with the [I(4-Etpy)_2_]PF_6_ complex as a minor solvate in the channels of polymerically packed [Ag(bpyMe_2_)_2_]PF_6_ complexes connected by argentophilic interactions (Ag^+^—Ag^+^ distances of 3.444(1), 3.456(2), 3.590(2), 3.735(2), and 3.866(1) Å), with a crystallographic Ag^+^:I^+^ ratio of 5:1. Serendipitously, this is the first time [I(4-Etpy)_2_]^+^ has been observed in the solid state as it proved unobtainable and defied crystallization as the pure species, possibly due to decomposition when concentrated, which is a necessary process of any solution-based crystallization method. The I^+^–N bond distances were 2.25(1) and 2.26(1) Å, which are within the expected range based on other previously reported examples of halonium ions,^11,25^ and therefore warrant no further comment.

**Figure 4:**
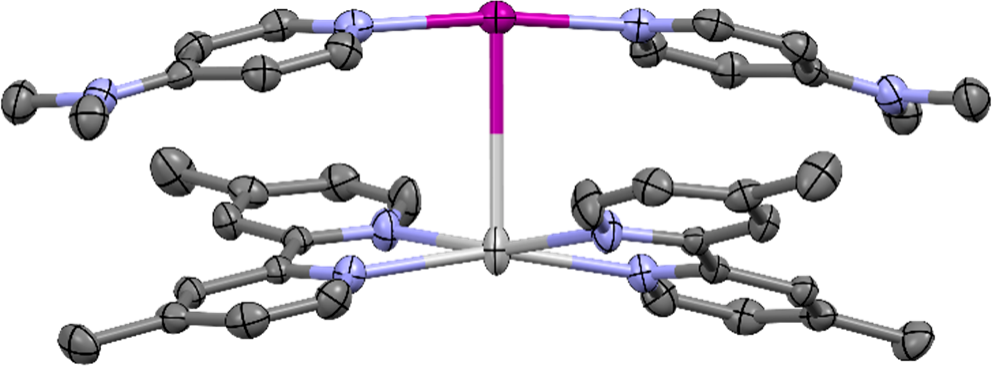
Single-crystal X-ray structure of the pair of complexes **12**, showing the I^+^—Ag^+^ (3.4043(4) Å) interaction (thermal ellipsoids at 50% probability; PF_6_ anions and hydrogen atoms omitted for clarity). Color key: purple = iodine, light gray = silver, blue = nitrogen, dark gray = carbon.

In addition to the first literature example in the pair of complexes **7**, a second example of an I^+^ ion acting as a nucleophile was successfully observed for the pair of complexes **12** (Figure 4). It should be noted that the success of **7** and **12** is consistent with ^15^N NMR observations (*vide supra*), so the labor-intensive practice of testing the unit cells of a statistically valid number of crystals in ultimately unsuccessful combinations of potential cocrystals could possibly be replaced; instead, the easier and more indicative process of ^15^N NMR screening could be used to select combinations that have a higher chance of success prior to attempts to more definitively confirm this interaction in the solid state.

**Figure 5:**
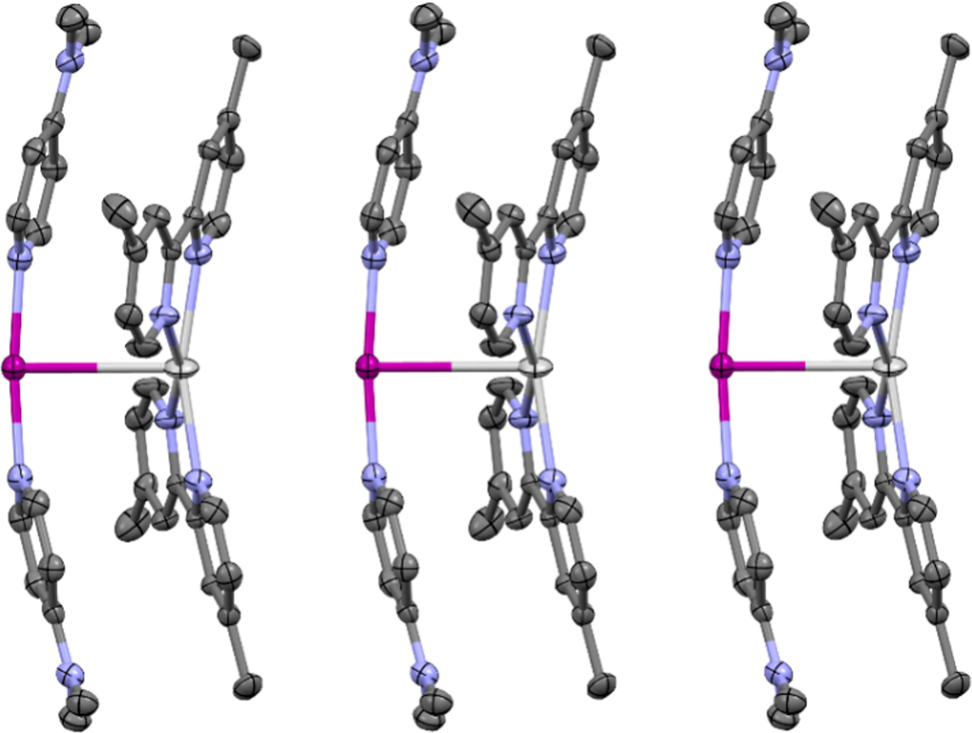
Packing of three molecules of the pair of complexes **12**, showing the second, longer I^+^···Ag^+^ (3.8005(4) Å) spacing observed between the pairs of complexes in their solid-state packing (thermal ellipsoids at 50% probability; PF_6_ anions and hydrogen atoms omitted for clarity). Color key: purple = iodine, light gray = silver, blue = nitrogen, dark gray = carbon.

The solid-state structure of the pair of complexes **12** exhibits the second and shortest example of an iodonium complex acting as a nucleophile, with an I^+^···Ag^+^ distance of 3.4043(4) Å (*cf.* 3.4608(3) Å for **7**). Unlike in **7** where the structural details closely resembled their individual I^+^ and Ag^+^ components, the structure of the [I(4-DMAP)_2_]^+^ component in **12** was observed to have significantly changed to accommodate the I^+^···Ag^+^ interaction. This is most obviously apparent in the loss of coplanarity of the two 4-DMAP ligands, with a 32.9° angle between the two NC_5_ planes of the 4-DMAP aromatic rings (*cf*. the 3.6° angle between the two NC_5_ planes for pure [I(4-DMAP)_2_]PF_6_). The pair of cations of **12**, [I(4-DMAP)_2_]^+^ and [Ag(bpyMe_2_)_2_]^+^, pack in an alternating polymeric fashion creating a second, but longer, I^+^···Ag^+^ distance of 3.8005(4) Å (Figure 5), which is noticeably longer than the van der Waals radii of the respective atoms (*cf*. van der Waals radii of silver + iodine = 3.70 Å). This polymeric array of alternating I^+^/Ag^+^ units creates continuous off-center π-stacking interactions between the 4-DMAP and bpyMe_2_ ligands, with centroid–centroid distances of the NC_5_ aromatic rings of 3.599 and 3.610 Å, similar to those observed in **7** (centroid–centroid distances between NC_5_ and N_3_C_2_ rings = 3.540, 3.675, 3.738, 3.771 Å). The I^+^–N (2.251(3) Å; a crystallographic symmetry operation generates half of the cocrystal structure) and Ag^+^–N (2.343(2) and 2.358(3) Å) bond lengths of **12** showed no crystallographically distinguishable difference from the solid-state structures of their pure individual components [I(4-DMAP)_2_]PF_6_ (I^+^–N = 2.236(3), 2.251(3) Å) and [Ag(bpyMe_2_)_2_]PF_6_ (**13_1**: Ag^+^–N range = 2.265(2)–2.400(2) Å; **13_2**: Ag^+^–N range = 2.251(8)–2.42(1) Å).

While there is only one prior example of a halonium ion acting as a nucleophile, there are examples of *neutral* iodine atoms donating to silver(I). In the literature, six solid-state examples were found of (R)I–Ag^+^ interactions, with bond lengths ranging from 2.8354(8) to 3.2877(6) Å,^26−29^ though only two of those examples contain comparable motifs: one based on a substituted iodophenyl group as the donor of the neutral iodine (3.2877(6) Å)^26^ and the other with a substituted iodopyrimidine group as the donor (3.1875(8) Å).^27^

**Figure 6:**
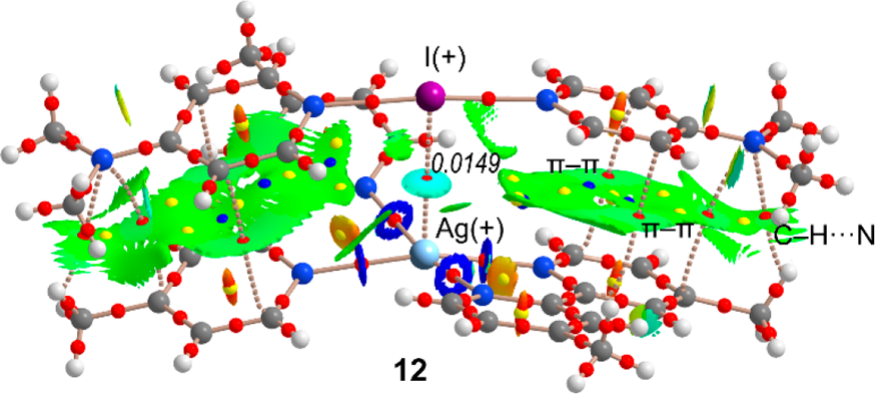
Distribution of bond, ring, and cage critical points (red, yellow, and blue spheres, respectively) and bond paths (dashed ones correspond to noncovalent interactions) for complex **12** at the M06-2X/def2-QZVP level of theory. The NCIplot surface (isosurface = 0.5 au) is also represented superimposed to the QTAIM. The color scale is −0.04 < sign(λ_2_)ρ < 0.04 au.

The intriguing solid-state structure of compound **12** has been further analyzed by DFT calculations in order to shed light on the nature of the I^+^···Ag^+^ interaction. The geometry of the fully optimized compound **12** is given in Figure S32, showing a geometry that is similar to the one observed experimentally. It also shows a marked loss of coplanarity of the two 4-DMAP ligands and the counterintuitive I^+^···Ag^+^ interaction, which is maintained in the isolated dimer. The QTAIM analysis combined with the NCIPlot is represented in Figure 6, where the X-ray geometry has been used. The distribution of critical points (CPs) and bond paths confirms the coexistence of the I^+^···Ag^+^ interaction and π-stacking forces. That is, a bond CP (red sphere) and bond path interconnect the Ag and I atoms. Moreover, the π-stacking is characterized by three bond CPs that interconnect two C atoms and the exocyclic N atom of each 4-DMAP ligand to three C atoms of the bpyMe_2_ ligands. Finally, the QTAIM analysis also reveals a weak C–H···N contact between one C–H bond of the methyl group of bpyMe_2_ ligand and the N atom of the NMe_2_ group of 4-DMAP. Further analysis of the π-stacking interaction and its contribution to the stabilization of the assembly is given in the ESI (see Figure S33). It is interesting to highlight that the NCIPlot (Figure 6) shows a well-defined blue isosurface located between the I and Ag atoms, thus confirming the attractive nature of the I^+^···Ag^+^ interaction. The value of the charge density (ρ) at the bond CP is 0.0149 au, which is slightly greater than that reported recently for a similar complex (0.0130 au),^20^ thus suggesting a stronger interaction. Other parameters at the bond CP are also useful to analyze the I^+^···Ag^+^ interaction. In particular, the positive value of the Laplacian of ρ (∇^2^ρ = 0.0394 au) combined with the negligible value of the total energy density (H = +0.0001 au) indicates that the interaction has a marked noncovalent character.

**Figure 7:**
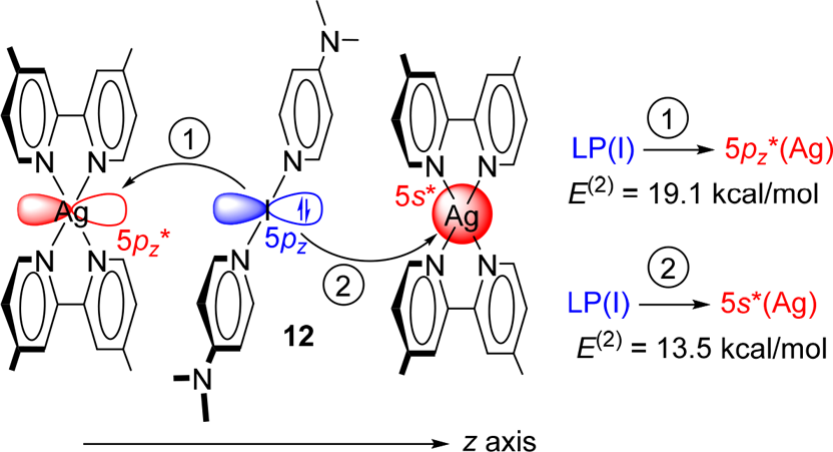
Representation of the orbital donor–acceptor interactions in compound **12** obtained using the M06-2X/def2-QZVP wave function.

Finally, to investigate the I^+^···Ag^+^ from the perspective of a donor–acceptor interaction, the NBO^30^ analysis which focused on the second order perturbation analysis has been performed for compound **12**. This method has been recently used to investigate the energy stabilization associated with the donor–acceptor interactions in a similar system.^20^ The energetic results derived from the NBO calculations (Figure 7) reveal that in the dimer of compound **12**, two relevant orbital donor–acceptor contributions exist. In both, the electron donation comes from one of the free lone pairs of I^+^ (located at atomic orbital 5*p*_*z*_) to (i) the empty 5*s* orbital of Ag(2) and (ii) the empty 5*p*_*z*_ orbital of Ag^+^. The energy stabilization of the system due to both donor–acceptor interactions is −32.6 kcal/mol. This result confirms the nucleophilic character of I^+^ and the electrophilic character of Ag^+^ and the donor–acceptor nature of the interaction that largely contributes to the stabilization of the system that compensates for the electrostatic repulsion between the formal positive charges. Further support for this explanation is provided by the analysis of the frontier molecular orbitals of the isolated [I(4-DMAP)_2_]^+^ fragment, which shows the significant participation of the *p*_*z*_ atomic orbital of I in the HOMO (highest occupied molecular orbital), as detailed in Figure S34. Finally, a bond order of 0.16 has been obtained for the Ag···I contact using the Wiberg bond index (WBI),^31^ which confirms the existence and noncovalent nature of this interaction. The WBI corresponding to the Ag···I interaction in **12** is similar to those reported for argentophilic interactions.^32^

## Conclusions

The new three-coordinate silver(I) complexes (**2**–**6**) were synthesized, and their utility in the strategy of *partial* cation exchange has been demonstrated to generate a series of the related pairs of complexes (**8**–**12**). These partial cation exchanges upon reaction with 0.5 equiv of elemental iodine were monitored by ^1^H and ^15^N NMR studies and found to proceed quantitatively in all cases. Closer inspection of the ^15^N NMR resonances revealed that the silver(I) component in the pair of complexes **12** had shifted relative to its ^15^N NMR value of the pure complex, indicating the observation for the second time of an I^+^···Ag^+^ interaction. This interaction was further confirmed in the solid state by single-crystal X-ray diffraction with an I^+^···Ag^+^ distance of 3.4043(4) Å, significantly shorter than the only other known example of this “nucleophilic” iodonium interaction.

The interaction was interrogated computationally revealing the nucleophilic nature of the I^+^ that uses one of its available lone pairs to donate charge to the electrophilic Ag^+^, as demonstrated by the second order perturbation analysis. The QTAIM analysis confirmed the existence of the I^+^···Ag^+^ contact (bond CP and bond path connecting both atoms), and the NCIplot revealed its attractive nature, in spite of the electrostatic repulsion between the positive charges.

## Experimental Section

### General Considerations

All reagents and solvents were obtained from commercial suppliers and used without further purification. For structural NMR assignments, ^1^H NMR spectra were recorded on a Bruker Avance 300 MHz spectrometer at 25 °C in CD_2_Cl_2_. The ^1^H–^15^N NMR correlation spectra were recorded on a Bruker Avance III 500 MHz spectrometer at 25 °C in CD_2_Cl_2_, and in the instances of complexes containing the mtz or 4-DMAP ligands which possess multiple independent nitrogen environments, only the values for the nitrogen atoms of interest (i.e., those that are directly bonded to the Ag^+^ or I^+^ ions) are reported. Chemical shifts are reported on the δ scale in ppm using the residual solvent signal as internal standard (CD_2_Cl_2_; δ_H_ 5.32) or, for ^1^H–^15^N NMR spectroscopy, to an external *d*_3_-MeNO_2_ standard. For ^1^H NMR spectroscopy, each resonance was assigned according to the following conventions: chemical shift (δ) measured in ppm, observed multiplicity, number of hydrogens, observed coupling constant (J Hz), and assignment. Multiplicities are denoted as s (singlet), d (doublet), t (triplet), q (quartet), m (multiplet), and br (broad).

The single-crystal X-ray data for **2**, **4**, **6**, and **7_2** were collected at 170 K using a Bruker-Nonius Kappa CCD diffractometer with an APEX-II detector with graphite-monochromatized Mo–Kα (λ = 0.71073 Å) radiation. The program COLLECT^33^ was used for the data collection, and the DENZO/SCALEPACK^34^ program was used for the data reduction. The single-crystal X-ray data for **3**, **7_1**, **8**, and **9** and for **5** were collected at 120 K and 273 K, respectively, due to a catastrophic phase change being observed at lower temperatures, using an Agilent SuperNova dual wavelength diffractometer with an Atlas detector using mirror-monochromated Cu–Kα (λ = 1.54184 Å) radiation. The program CrysAlisPro^35^ was used for the data collection and reduction on the SuperNova diffractometer, and the intensities were absorption corrected using a Gaussian face index absorption correction method. All structures were solved by intrinsic phasing (SHELXT)^36^ and refined by full-matrix least-squares on *F*^2^ using the OLEX2,^37^ utilizing the SHELXL-2015 module.^38^ Anisotropic displacement parameters were assigned to non-H atoms and isotropic displacement parameters for all H atoms and were constrained to multiples of the equivalent displacement parameters of their parent atoms with *U*_iso_(H) = 1.2*U*_eq_ (parent atom). The X-ray single-crystal data and CCDC numbers of all new structures are included below.

The ^1^H and ^15^N NMR data of [Ag(bpy)_2_]PF_6_,^20^ [Ag(bpy)(mtz)]PF_6_ (**1**),^20^ [I(mtz)_2_]PF_6_,^20^ [I(4-Etpy)_2_]PF_6_,^25^ and [I(4-DMAP)_2_]PF_6_^25^ have been previously reported, and similarly, the solid-state structures (where applicable) were also obtained from these same literature sources.

The preparation of all three-coordinate silver(I) complexes was performed in the absence of light sources, both natural and artificial, to avoid accidental decomposition in solution.

#### Preparation of [Ag(bpyMe_2_)(mtz)]PF_6_ (**2**)

An MeCN (acetonitrile) (5 mL) suspension of bpyMe_2_ (73.7 mg, 0.4 mmol) was added to a stirred MeCN (5 mL) solution of AgPF_6_ (101.1 mg, 0.4 mmol), and after 5 min, mtz (28.4 μL mg, 0.4 mmol) was added neat. The reaction was stirred for 30 min, and then all volatiles were removed under reduced pressure to leave a white solid. Recovered yield = 183.2 mg (0.352 mmol, 88%). Crystals suitable for single-crystal X-ray diffraction were obtained from DCM (dichloromethane) vapor diffused with pentane. ^1^H NMR (300 MHz, CD_2_Cl_2_) δ 8.56 (d, *J* = 5.1 Hz, 2H), 8.04 (s, 2H), 7.95 (s, 2H), 7.43 (s, 1H), 7.41 (s, 1H), 4.24 (s, 3H), 2.56 (s, 6H). ^15^N NMR (500 MHz, CD_2_Cl_2_) δ −18.65, −81.26, −119.22, −141.63. Analysis Found: C, 34.53; H, 3.03: N, 12.89%. Calculated for C_15_H_17_AgF_6_N_5_P: C, 34.64; H, 3.29: N, 13.46%.

#### Preparation of [Ag(bpy)(4-Etpy)]PF_6_ (**3**)

An MeCN (5 mL) solution of bpy (62.5 mg, 0.4 mmol) was added to a stirred MeCN (5 mL) solution of AgPF_6_ (101.1 mg, 0.4 mmol), and after 60 s, 4-Etpy (45.5 μL mg, 0.4 mmol) was added neat. The reaction was stirred for 30 min, and then all volatiles were removed under reduced pressure to leave a pale yellow solid. Recovered yield = 185.0 mg (0.358 mmol, 90%). Crystals suitable for single-crystal X-ray diffraction were obtained from DCM vapor diffused with pentane. ^1^H NMR (300 MHz, CD_2_Cl_2_) δ 8.72 (d, *J* = 4.3 Hz, 2H), 8.52 (d, *J* = 6.4 Hz, 2H), 8.27 (d, *J* = 8.1 Hz, 2H), 8.11 (td, *J* = 7.9, 1.6 Hz, 2H), 7.63 (ddd, *J* = 7.5, 5.1, 0.9 Hz, 2H), 7.45 (d, *J* = 6.3 Hz, 2H), 2.79 (q, *J* = 7.5 Hz, 2H), 1.32 (t, *J* = 7.6 Hz, 3H). ^15^N NMR (500 MHz, CD_2_Cl_2_) δ −110.10, −132.55. Analysis Found: C, 38.73; H, 3.18: N, 8.34%. Calculated for C_17_H_17_AgF_6_N_3_P·0.3(H_2_O): C, 39.15; H, 3.40: N, 8.06%.

#### Preparation of [Ag(bpyMe_2_)(4-Etpy)]PF_6_ (**4**)

An MeCN (5 mL) suspension of bpyMe_2_ (73.7 mg, 0.4 mmol) was added to a stirred MeCN (5 mL) solution of AgPF_6_ (101.1 mg, 0.4 mmol), and after 5 min, 4-Etpy (45.5 μL mg, 0.4 mmol) was added neat. The reaction was stirred for 30 min, and then all volatiles were removed under reduced pressure to leave a yellow crystalline solid. Recovered yield = 194.9 mg (0.358 mmol, 90%). Crystals suitable for single-crystal X-ray diffraction were obtained from DCM vapor diffused with diisopropyl ether. ^1^H NMR (300 MHz, CD_2_Cl_2_) δ 8.60–8.45 (m, 4H), 8.06 (s, 2H), 7.49–7.38 (m, 4H), 2.79 (q, *J* = 7.0 Hz, 2H), 2.56 (s, 6H), 1.31 (t, *J* = 7.5 Hz, 3H). ^15^N NMR (500 MHz, CD_2_Cl_2_) δ −117.70, −130.76. Analysis Found: C, 41.54; H, 3.87: N, 8.07%. Calculated for C_19_H_21_AgF_6_N_3_P: C, 41.93; H, 3.89: N, 7.72%.

#### Preparation of [Ag(bpy)(4-DMAP)]PF_6_ (**5**)

An MeCN (5 mL) solution of bpy (62.5 mg, 0.4 mmol) was added to a stirred MeCN (5 mL) solution of AgPF_6_ (101.1 mg, 0.4 mmol), and after 60 s, a MeCN (1 mL) solution of 4-DMAP (48.9 mg, 0.4 mmol) was added. The reaction was stirred for 30 min, during which time the colorless solution had become an orange/yellow color. All volatiles were removed under reduced pressure to leave a khaki solid. Recovered yield = 193.9 mg (0.365 mmol, 91%). Crystals suitable for single-crystal X-ray diffraction were obtained from DCM vapor diffused with pentane. ^1^H NMR (300 MHz, CD_2_Cl_2_) δ 8.70 (d, *J* = 4.8 Hz, 2H), 8.27 (d, *J* = 8.1 Hz, 2H), 8.15–8.06 (m, 4H), 7.61 (dd, *J* = 7.0, 5.4 Hz, 2H), 6.64 (d, *J* = 6.9 Hz, 2H), 3.09 (s, 6H). ^15^N NMR (500 MHz, CD_2_Cl_2_) δ −108.73, −169.62, −314.41. Analysis Found: C, 38.21; H, 3.39: N, 10.76%. Calculated for C_17_H_18_AgF_6_N_4_P: C, 38.44; H, 3.42: N, 10.55%.

#### Preparation of [Ag(bpyMe_2_)(4-DMAP)]PF_6_ (**6**)

An MeCN (5 mL) suspension of bpyMe_2_ (73.7 mg, 0.4 mmol) was added to a stirred MeCN (5 mL) solution of AgPF_6_ (101.1 mg, 0.4 mmol), and after 5 min, a MeCN (1 mL) solution of 4-DMAP (48.9 mg, 0.4 mmol) was added. The reaction was stirred for 30 min, during which time the colorless solution had become an orange/yellow color. All volatiles were removed under reduced pressure to leave a beige solid. Recovered yield = 189.3 mg (0.338 mmol, 85%). Crystals suitable for single-crystal X-ray diffraction were obtained from DCM vapor diffused with pentane. ^1^H NMR (300 MHz, CD_2_Cl_2_) δ 8.52 (d, J = 5.2 Hz, 2H), 8.08 (dd, *J* = 5.6, 1.4 Hz, 2H), 8.05 (s, 2H), 7.40 (d, *J* = 4.6 Hz, 2H), 6.64 (dd, *J* = 5.6, 1.5 Hz, 2H), 3.09 (s, 6H), 2.56 (s, 6H). ^15^N NMR (500 MHz, CD_2_Cl_2_) δ −116.49, −169.95, −314.18. Analysis Found: C, 40.89; H, 3.92: N, 10.26%. Calculated for C_19_H_22_AgF_6_N_4_P: C, 40.81 H, 3.97: N, 10.02%.

### Formation of the Ag^+^/I^+^ Pairs of Complexes (**8**–**12**)

A general procedure for the conversion of the three-coordinate silver(I) complexes (**1**–**6**) to iodonium ions (as the pairs of complexes **8**–**12**) was followed: a CD_2_Cl_2_ (0.5 mL) solution of the three-coordinate complexes (**1**–**6**, 0.01 mmol) and a CD_2_Cl_2_ (0.5 mL) solution of I_2_ (1.3 mg, 0.005 mmol) were combined to immediately generate a yellow precipitate (AgI). The reactions were stirred for 10 min and filtered, and their NMR spectra were recorded. *N.b*.: ^1^H and ^15^N NMR data for the pair of complexes **7** matched that previously reported for this combination in the literature.

#### Pair of Complexes **8**

^1^H NMR (500 MHz, CD_2_Cl_2_) δ 8.47 (br. s, 4H), 8.08 (br. s, 4H), 7.98 (s, 2H), 7.92 (s, 2H), 7.40 (br. s, 4H), 4.25 (s, 6H), 2.56 (br. s, 12H). ^15^N NMR (500 MHz, CD_2_Cl_2_) δ −20.03, −137.97, −142.21 (a resonance for the bpyMe_2_ nitrogen atoms was not observed).

#### Pair of Complexes **9**

^1^H NMR (300 MHz, CD_2_Cl_2_) δ 8.64 (d, *J* = 4.2 Hz, 4H), 8.58 (dd, *J* = 5.2, 1.4 Hz, 4H), 8.29 (d, *J* = 8.1 Hz, 4H), 8.09 (td, *J* = 7.9, 1.7 Hz, 4H), 7.58 (ddd, *J* = 7.6, 5.0, 1.1 Hz, 4H), 7.41 (d, *J* = 6.5 Hz, 4H), 2.83 (q, *J* = 7.6 Hz, 4H), 1.30 (t, *J* = 7.6 Hz, 6H). ^15^N NMR (500 MHz, CD_2_Cl_2_) δ −105.82, −181.61.

#### Pair of Complexes **10**

^1^H NMR (300 MHz, CD_2_Cl_2_) δ 8.58 (d, *J* = 6.6 Hz, 4H), 8.44 (d, *J* = 5.2 Hz, 4H), 8.06 (s, 4H), 7.39 (dd, *J* = 12.0, 5.5 Hz, 8H), 2.83 (q, *J* = 7.6 Hz, 4H), 2.56 (s, 12H), 1.30 (t, *J* = 7.6 Hz, 6H). ^15^N NMR (500 MHz, CD_2_Cl_2_) δ −114.10, −181.74.

#### Pair of Complexes **11**

^1^H NMR (300 MHz, CD_2_Cl_2_) δ 8.64 (d, *J* = 4.9 Hz, 4H), 8.28 (d, *J* = 8.1 Hz, 4H), 8.13–8.03 (m, 8H), 7.58 (ddd, *J* = 7.3, 4.9, 0.8 Hz, 4H), 6.50 (d, *J* = 7.3 Hz, 4H), 3.11 (s, 12H). ^15^N NMR (500 MHz, CD_2_Cl_2_) δ −105.81, −216.13.

#### Pair of Complexes **12**

^1^H NMR (300 MHz, CD_2_Cl_2_) δ 8.44 (d, *J* = 5.1 Hz, 4H), 8.09–8.03 (m, 4H), 7.36 (d, *J* = 4.3 Hz, 4H), 6.50 (d, *J* = 7.3 Hz, 4H), 3.10 (s, 12H), 2.55 (s, 12H). ^15^N NMR (500 MHz, CD_2_Cl_2_) δ −112.14, −216.01.

#### Preparation of [Ag(bpyMe_2_)_2_]PF_6_ (**13**)

A DCM (2 mL) solution of bpyMe_2_ (73.7 mg, 0.4 mmol) was added to a stirred MeCN (1 mL) solution of AgPF_6_ (50.6 mg, 0.2 mmol) to give a pale yellow solution. The solution was stirred for 30 min and then left to evaporate to dryness to give the product as a yellow solid in quantitative yield. Crystals suitable for single-crystal X-ray diffraction were obtained from partial evaporation of a DCM/MeCN (3:1 ratio) solution (**13_1**) and from DCM vapor diffused with diisopropyl ether (**13_2**). ^1^H NMR (300 MHz, CD_2_Cl_2_) δ 8.43 (d, *J* = 5.2 Hz, 4H), 8.05 (s, 4H), 7.37 (d, *J* = 4.5 Hz, 4H), 2.55 (s, 12H).). ^15^N NMR (500 MHz, CD_2_Cl_2_) δ −114.35. Analysis Found: C, 46.52; H, 3.96: N, 9.07%. Calculated for C_24_H_24_AgF_6_N_4_P: C, 46.40; H, 3.89: N, 9.02%.

## Supporting Information Available

Additional experimental details, molecular structures, crystallographic details, ^15^N NMR comparison tables, NMR spectra, and details of quantum chemical calculations (PDF)

## Accession Codes

CCDC 2062093–2062101 contain the supplementary crystallographic data for this paper. These data can be obtained free of charge via www.ccdc.cam.ac.uk/data_request/cif, or by emailing data_request@ccdc.cam.ac.uk, or by contacting The Cambridge Crystallographic Data Centre, 12 Union Road, Cambridge CB2 1EZ, UK; fax: +44 1223 336033.
