# Supplementary material for: Utility of Three-Coordinate Silver Complexes Toward the Formation of Iodonium Ions
Source: Inorg Chem. 2021 Mar 25;60(7):5383–90. doi: 10.1021/acs.inorgchem.1c00409 (PMC8154410; doi:10.1021/acs.inorgchem.1c00409)
Supplement: Supplementary file 1 — ic1c00409_si_001.pdf [file ic1c00409_si_001.pdf]

The Utility of 3-coordinate Silver Complexes Toward the Formation of Iodonium Ions

Supporting Information

Jas S. Ward,[a] Antonio Frontera,[b] Kari Rissanen[a]**

[a] University of Jyvaskyla, Department of Chemistry, Jyväskylä 40014, Finland.

[b] Department of Chemistry, Universitat de les Illes Balears, Crts de Valldemossa km 7.6, 07122 Palma de Mallorca (Baleares), Spain.

E-mail: james.s.ward@jyu.fi, kari.t.rissanen@jyu.fi

Contents

Synthesis S2
General Considerations S2
Synthesis of Silver(I) Complexes (**1-6**) S4
Formation of Ag^+/I^+ Pairs of Complexes (**8-12**) S10
Comparison Tables of ^{15}N NMR Resonances for Ag^+–N and I^+–N S16
NMR Spectra S18
Computational Details S40
References S43

Synthesis

General Considerations

All reagents and solvents were obtained from commercial suppliers and used without further purification. For structural NMR assignments, ^{1}H NMR spectra were recorded on a Bruker Avance 300 MHz spectrometer at 25ºC in CD_2Cl_2. The ^{1}H-^{15}N NMR correlation spectra were recorded on a Bruker Avance III 500 MHz spectrometer at 25ºC in CD_2Cl_2, and in the instances of complexes containing the mtz or 4-DMAP ligands which possess multiple independent nitrogen environments, only the values for the nitrogen atoms of interest (*i.e.*, those that are directly bonded to the Ag^+ or I^+ ions) are reported. Chemical shifts are reported on the δ scale in ppm using the residual solvent signal as internal standard (CD_2Cl_2; δ_H 5.32), or for ^{1}H-^{15}N NMR spectroscopy, to an external d_3-$MeNO_2$ standard. For ^{1}H NMR spectroscopy, each resonance was assigned according to the following conventions: chemical shift (δ) measured in ppm, observed multiplicity, number of hydrogens, observed coupling constant (*J* Hz), and assignment. Multiplicities are denoted as: s (singlet), d (doublet), t (triplet), q (quartet) m (multiplet) and br (broad).

The single crystal X-ray data for **2**, **4**, **6**, and **7_2** were collected at 170 K using Bruker-Nonius Kappa CCD diffractometer with an APEX-II detector with graphite-monochromatised Mo-Kα (λ = 0.71073 Å) radiation. The program COLLECT[1] was used for the data collection and DENZO/SCALEPACK[2] for the data reduction. The single crystal X-ray data for **3**, **7_1**, **8**, and **9** were collected at 120 K, and **5** at 273 K due to a catastophic phase change being observed at lower temperatures, using an Agilent SuperNova dual wavelength diffractometer with an Atlas detector using mirror-monochromated Cu-Kα (λ = 1.54184 Å) radiation. The program CrysAlisPro[3] was used for the data collection and reduction on the SuperNova diffractometer, and the intensities were absorption corrected using a gaussian face index absorption correction method. All structures

were solved by intrinsic phasing (SHELXT)[4] and refined by full-matrix least squares on F^2 using the OLEX2,[5] utilizing the SHELXL-2015 module.[6] Anisotropic displacement parameters were assigned to non-H atoms and isotropic displacement parameters for all H atoms were constrained to multiples of the equivalent displacement parameters of their parent atoms with $U_{iso}(H) = 1.2\ U_{eq}$ (parent atom). The X-ray single crystal data and CCDC numbers of all new structures are included below.

The ^{1}H and ^{15}N NMR data of $[Ag(bpy)_2]PF_6$,[7] $[Ag(bpy)(mtz)]PF_6$ (**1**),[7] $[I(mtz)_2]PF_6$,[7] $[I(4\text{-Etpy})_2]PF_6$,[8] and $[I(4\text{-DMAP})_2]PF_6$[8] have been previously reported, and similarly the solid-state structures (where applicable) were also obtained from these same literature sources.

The following abbreviations are used: mtz = 1-methyl-1H-1,2,3-triazole, 4-Etpy = 4-ethylpyridine, 4-DMAP = N,N-dimethylpyridin-4-amine, bpy = 2,2′-bipyridyl, $bpyMe_2$ = 4,4′-dimethyl-2,2′-dipyridyl, DCM = dichloromethane, MeCN = acetonitrile.

Synthesis of Silver(I) Complexes (**1-6**)

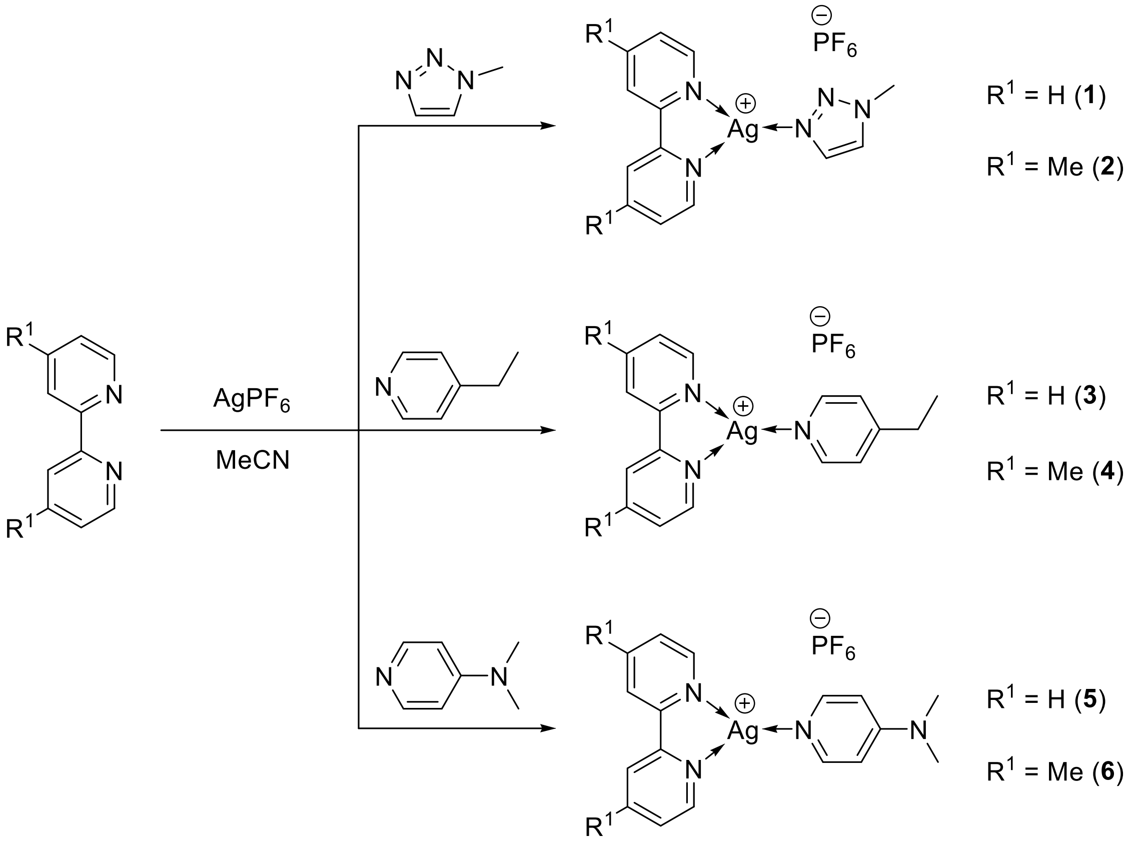

The preparation of all 3-coordinate silver(I) complexes was performed in the absence of light sources, both natural and artificial, to avoid accidental decomposition in solution.

Preparation of $[Ag(bpyMe_2)(mtz)]PF_6$ (**2**): An MeCN (5 mL) suspension of $bpyMe_2$ (73.7 mg, 0.4 mmol) was added to a stirred MeCN (5 mL) solution of $AgPF_6$ (101.1 mg, 0.4 mmol), and after 5 minutes mtz (28.4 µL mg, 0.4 mmol) was added neat. The reaction was stirred for 30 minutes, then all volatiles were removed under reduced pressure to leave a white solid. Recovered yield = 183.2 mg (0.352 mmol, 88%). Crystals suitable for single crystal X-ray diffraction were obtained from DCM vapor diffused with pentane. ^1H NMR (300 MHz, CD_2Cl_2) δ 8.56 (d, J = 5.1 Hz, 2H), 8.04 (s, 2H), 7.95 (s, 2H), 7.43 (s, 1H), 7.41 (s, 1H), 4.24 (s, 3H), 2.56 (s, 6H). ^{15}N NMR (500 MHz, CD_2Cl_2) δ -18.65, -81.26, -119.22, -141.63. Analysis Found: C, 34.53; H, 3.03: N, 12.89%. Calculated for $C_{15}H_{17}AgF_6N_5P$: C, 34.64; H, 3.29: N, 13.46%. Crystal data for **2**: CCDC-2062093, $[C_{15}H_{17}AgN][PF_6]\cdot 0.5(CH_2Cl_2)$, M = 1125.28, colorless needle, 0.08 x 0.09 x 0.40 mm^3, monoclinic, space group $C2/c$, a = 15.9672(3) Å, b = 19.3029(3) Å, c = 13.7177(3) Å, β = 101.058(1)°, V = 4149.48(14) $Å^3$, Z = 4, D_{calc} = 1.801 gcm^{-3}, F000 = 2232, μ = 1.24 mm^{-1}, T = 170(1) K, θ_{max} = 27.9°, 4582 total reflections, 3580 with $I_o > 2\sigma(I_o)$, R_{int} = 0.038, 4582 data, 270 parameters, no restraints, GooF = 1.04, 0.95 <dΔρ < -0.81 $eÅ^{-3}$, $R[F^2 > 2\sigma(F^2)]$ = 0.052, $wR(F^2)$ = 0.129.

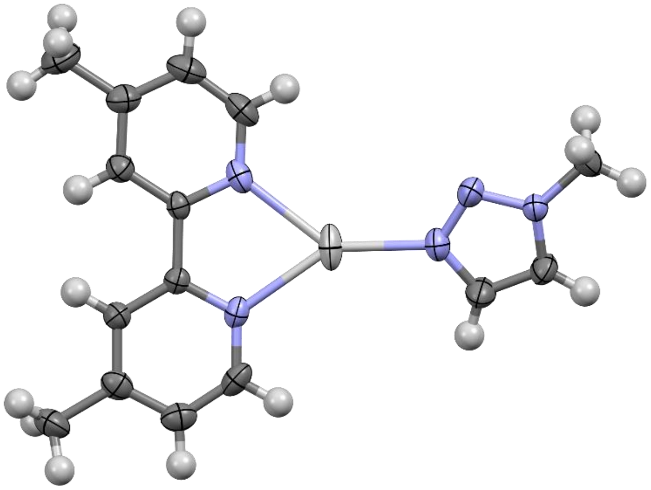

Figure S1: The crystal structure of ***2*** *(thermal ellipsoids at 50%probability; PF_6 anion and solvates omitted for clarity).*

Preparation of [Ag(bpy)(4-Etpy)]PF_6 (**3**): An MeCN (5 mL) solution of bpy (62.5 mg, 0.4 mmol) was added to a stirred MeCN (5 mL) solution of $AgPF_6$ (101.1 mg, 0.4 mmol), and after 60 seconds 4-Etpy (45.5 µL mg, 0.4 mmol) was added neat. The reaction was stirred for 30 minutes, then all volatiles were removed under reduced pressure to leave a pale yellow solid. Recovered yield = 185.0 mg (0.358 mmol, 90%). Crystals suitable for single crystal X-ray diffraction were obtained from DCM vapor diffused with pentane. ^{1}H NMR (300 MHz, CD_2Cl_2) δ 8.72 (d, *J* = 4.3 Hz, 2H), 8.52 (d, *J* = 6.4 Hz, 2H), 8.27 (d, *J* = 8.1 Hz, 2H), 8.11 (td, *J* = 7.9, 1.6 Hz, 2H), 7.63 (ddd, *J* = 7.5, 5.1, 0.9 Hz, 2H), 7.45 (d, *J* = 6.3 Hz, 2H), 2.79 (q, *J* = 7.5 Hz, 2H), 1.32 (t, *J* = 7.6 Hz, 3H). ^{15}N NMR (500 MHz, CD_2Cl_2) δ -110.10, -132.55. Analysis Found: C, 38.73; H, 3.18: N, 8.34%. Calculated for $C_{17}H_{17}AgF_6N_3P\cdot0.3(H_2O)$: C, 39.15; H, 3.40: N, 8.06%. Crystal data for **3**: CCDC-2062094, $([C_{17}H_{17}AgN_3][PF_6])_2$, M = 1032.35, colorless plate, 0.01 x 0.18 x 0.23 mm^3, triclinic, space group *P*-1 (No. 2), a = 7.2898(5) Å, b = 11.0381(10) Å, c = 12.3283(13) Å, α = 77.764(8)°, β = 73.812(8)°, γ = 82.580(7)°, V = 928.43(15) Å^3, Z = 1, D_{calc} = 1.846 gcm^{-3}, F000 = 512, µ = 10.15 mm^{-1}, T = 120.0(1) K, θ_{max} = 76.6°, 3804 total reflections, 3389 with $I_o > 2\sigma(I_o)$, R_{int} = 0.036, 3804 data, 254 parameters, no restraints, GooF = 1.04, 0.89 <dΔρ < -1.08 eÅ^{-3}, $R[F^2 > 2\sigma(F^2)]$ = 0.038, $wR(F^2)$ = 0.100.

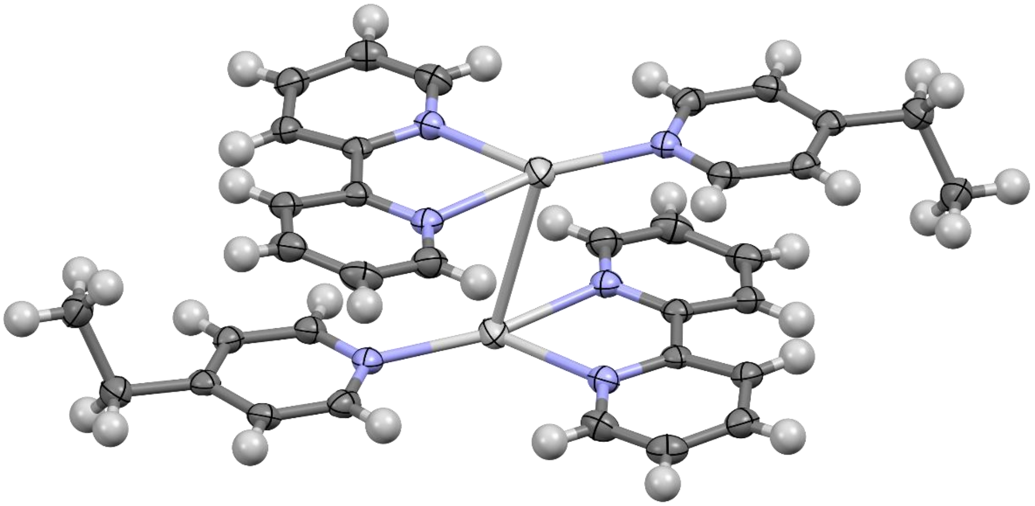

*Figure S2: The crystal structure of **3**, which was observed as a dimer (Ag^{+}---Ag^{+} = 3.1664(5) Å; thermal ellipsoids at 50% probability; PF_6 anions omitted for clarity).*

Preparation of $[Ag(bpyMe_2)(4\text{-}Etpy)]PF_6$ (**4**): An MeCN (5 mL) suspension of $bpyMe_2$ (73.7 mg, 0.4 mmol) was added to a stirred MeCN (5 mL) solution of $AgPF_6$ (101.1 mg, 0.4 mmol), and after 5 minutes 4-Etpy (45.5 µL mg, 0.4 mmol) was added neat. The reaction was stirred for 30 minutes, then all volatiles were removed under reduced pressure to leave a yellow crystalline solid. Recovered yield = 194.9 mg (0.358 mmol, 90%). Crystals suitable for single crystal X-ray diffraction were obtained from DCM vapor diffused with diisopropylether. ^1H NMR (300 MHz, CD_2Cl_2) δ 8.60 – 8.45 (m, 4H), 8.06 (s, 2H), 7.49 – 7.38 (m, 4H), 2.79 (q, J = 7.0 Hz, 2H), 2.56 (s, 6H), 1.31 (t, J = 7.5 Hz, 3H). ^{15}N NMR (500 MHz, CD_2Cl_2) δ -117.70, -130.76. Analysis Found: C, 41.54; H, 3.87: N, 8.07%. Calculated for $C_{19}H_{21}AgF_6N_3P$: C, 41.93; H, 3.89: N, 7.72%. Crystal data for **4**: CCDC-2062095, $([C_{19}H_{21}AgN_3][PF_6])_2$, M = 1088.45, colorless plate, 0.06 x 0.20 x 0.20 mm^3, monoclinic, space group $P2_1/c$, a = 14.9010(3) Å, b = 24.1489(7) Å, c = 12.9496(3) Å, β = 113.8390(10)°, V = 4262.26(18) $Å^3$, Z = 4, D_{calc} = 1.696 gcm^{-3}, F000 = 2176, µ = 1.08 mm^{-1}, T = 170(1) K, θ_{max} = 27.1°, 9385 total reflections, 5921 with $I_o > 2\sigma(I_o)$, R_{int} = 0.062, 9385 data, 587 parameters, 126 restraints, GooF = 1.05, 1.09 <dΔρ < -0.92 $eÅ^{-3}$, $R[F^2 > 2\sigma(F^2)]$ = 0.061, $wR(F^2)$ = 0.176.

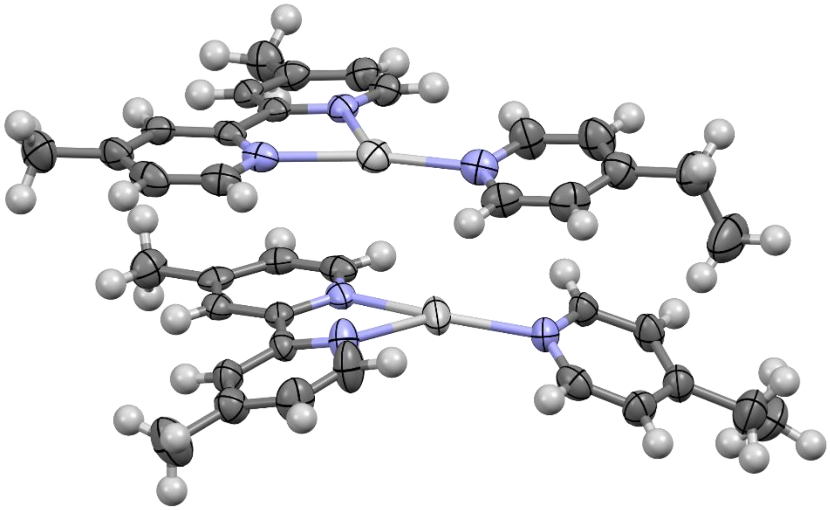

*Figure S3: The crystal structure of **4**, which was observed as a polymer (Ag^+---Ag^+ = 3.3314(7)/3.3526(8) Å; thermal ellipsoids at 50% probability; PF_6 anions omitted for clarity).*

Preparation of $[Ag(bpy)(4\text{-}DMAP)]PF_6$ (**5**): An MeCN (5 mL) solution of bpy (62.5 mg, 0.4 mmol) was added to a stirred MeCN (5 mL) solution of $AgPF_6$ (101.1 mg, 0.4 mmol), and after 60 seconds a MeCN (1 mL) solution of 4-DMAP (48.9 mg, 0.4 mmol) was added. The reaction was stirred for 30 minutes, during which time the colorless solution had become an orange/yellow color. All volatiles were removed under reduced pressure to leave a khaki solid. Recovered yield = 193.9 mg (0.365 mmol, 91%). Crystals suitable for single crystal X-ray diffraction were obtained from DCM vapor diffused with pentane. ^1H NMR (300 MHz, CD_2Cl_2) δ 8.70 (d, *J* = 4.8 Hz, 2H), 8.27 (d, *J* = 8.1 Hz, 2H), 8.15 – 8.06 (m, 4H), 7.61 (dd, *J* = 7.0, 5.4 Hz, 2H), 6.64 (d, *J* = 6.9 Hz, 2H), 3.09 (s, 6H). ^{15}N NMR (500 MHz, CD_2Cl_2) δ -108.73, -169.62, -314.41. Analysis Found: C, 38.21; H, 3.39: N, 10.76%. Calculated for $C_{17}H_{18}AgF_6N_4P$: C, 38.44; H, 3.42: N, 10.55%. Crystal data for **5**: CCDC-2062096, $([C_{17}H_{18}AgN_4][PF_6])_2$, M = 1062.39, colorless needle, 0.05 x 0.07 x 0.26 mm^3, monoclinic, space group $P2_1/c$, a = 7.3043(3) Å, b = 36.1675(14) Å, c = 15.6233(4) Å, β = 101.232(3)°, V = 4048.3(3) $Å^3$, Z = 4, D_{calc} = 1.743 gcm^{-3}, F000 = 2112, μ = 9.35 mm^{-1}, T = 273.0(1) K (due to catastophic phase changes observed when cooled to lower temperatures), θ_{max} = 76.2°, 5928 total reflections, 3924 with $I_o > 2\sigma(I_o)$, R_{int} = 0.065, 5928 data, 574 parameters, 117 restraints, GooF = 0.91, 0.45 <dΔρ < -0.33 $eÅ^{-3}$, $R[F^2 > 2\sigma(F^2)]$ = 0.041, $wR(F^2)$ = 0.104.

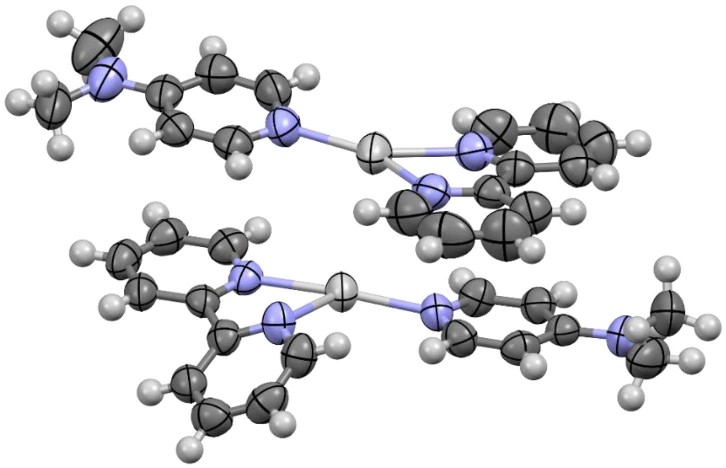

*Figure S4: The crystal structure of **5**, which was observed as a dimer (Ag^+---Ag^+ = 3.231(3) Å; thermal ellipsoids at 50% probability; PF_6 anions omitted for clarity).*

Preparation of [Ag(bpyMe$_2$)(4-DMAP)]PF$_6$ (**6**): An MeCN (5 mL) suspension of bpyMe$_2$ (73.7 mg, 0.4 mmol) was added to a stirred MeCN (5 mL) solution of AgPF$_6$ (101.1 mg, 0.4 mmol), and after 5 minutes a MeCN (1 mL) solution of 4-DMAP (48.9 mg, 0.4 mmol) was added. The reaction was stirred for 30 minutes, during which time the colorless solution had become an orange/yellow color. All volatiles were removed under reduced pressure to leave a beige solid. Recovered yield = 189.3 mg (0.338 mmol, 85%). Crystals suitable for single crystal X-ray diffraction were obtained from DCM vapor diffused with pentane. ^{1}H NMR (300 MHz, CD$_2$Cl$_2$) δ 8.52 (d, J = 5.2 Hz, 2H), 8.08 (dd, J = 5.6, 1.4 Hz, 2H), 8.05 (s, 2H), 7.40 (d, J = 4.6 Hz, 2H), 6.64 (dd, J = 5.6, 1.5 Hz, 2H), 3.09 (s, 6H), 2.56 (s, 6H). ^{15}N NMR (500 MHz, CD$_2$Cl$_2$) δ -116.49, -169.95, -314.18. Analysis Found: C, 40.89; H, 3.92: N, 10.26%. Calculated for $C_{19}H_{22}AgF_6N_4P$: C, 40.81 H, 3.97: N, 10.02%. Crystal data for **6**: CCDC-2062097, [$C_{19}H_{22}AgN_4$][PF$_6$], M = 559.24, colorless block, 0.06 x 0.12 x 0.26 mm^3, monoclinic, space group $P2_1/n$, a = 7.9456(2) Å, b = 15.8257(3) Å, c = 17.6523(5) Å, β = 102.5340(10)°, V = 2166.78(9) Å^3, Z = 4, D_{calc} = 1.714 gcm^{-3}, F000 = 1120, μ = 1.07 mm^{-1}, T = 170(1) K, θ_{max} = 27.1°, 4774 total reflections, 3321 with $I_o > 2\sigma(I_o)$, R_{int} = 0.047, 4774 data, 284 parameters, no restraints, GooF = 1.06, 0.62 <dΔρ < -0.74 eÅ^{-3}, $R[F^2 > 2\sigma(F^2)]$ = 0.045, $wR(F^2)$ = 0.136.

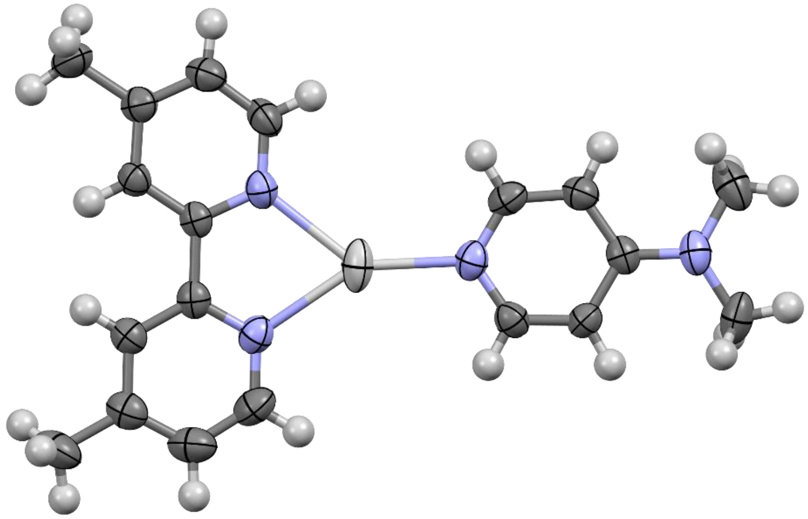

*Figure S5: The crystal structure of **6** (thermal ellipsoids at 50%probability; PF$_6$ anion omitted for clarity).*

Formation of Ag^+/I^+ Pairs of Complexes (**8-12**)

A general procedure for the conversion of the 3-coordinate silver(I) complexes (**1-6**) to iodonium ions (as Pairs of Complexes **8-12**) was followed: a CD_2Cl_2 (0.5 mL) solution of the 3-coordinate complexes (**1-6**, 0.01 mmol) and a CD_2Cl_2 (0.5 mL) solution of I_2 (1.3 mg, 0.005 mmol) were combined to immediately generate a yellow precipitate (AgI). The reactions were stirred for 10 minutes, filtered, and their NMR spectra recorded. N.B.: ^1H and ^{15}N NMR data for pair of complexes **7** matched that previously reported for this combination in the literature.

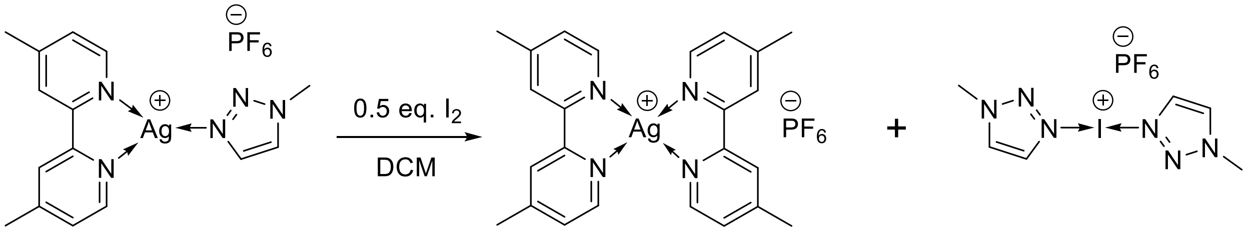

Pair of Complexes **8**: ^1H NMR (500 MHz, CD_2Cl_2) δ 8.47 (br. s, 4H), 8.08 (br. s, 4H), 7.98 (s, 2H), 7.92 (s, 2H), 7.40 (br. s, 4H), 4.25 (s, 6H), 2.56 (br. s, 12H). ^{15}N NMR (500 MHz, CD_2Cl_2) δ -20.03, -137.97, -142.21 (a resonance for the $bpyMe_2$ nitrogen atoms was not observed).

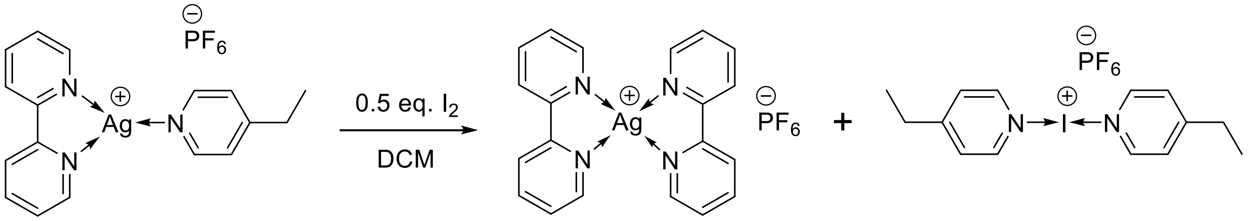

Pair of Complexes **9**: ^1H NMR (300 MHz, CD_2Cl_2) δ 8.64 (d, J = 4.2 Hz, 4H), 8.58 (dd, J = 5.2, 1.4 Hz, 4H), 8.29 (d, J = 8.1 Hz, 4H), 8.09 (td, J = 7.9, 1.7 Hz, 4H), 7.58 (ddd, J = 7.6, 5.0, 1.1 Hz, 4H), 7.41 (d, J = 6.5 Hz, 4H), 2.83 (q, J = 7.6 Hz, 4H), 1.30 (t, J = 7.6 Hz, 6H). ^{15}N NMR (500 MHz, CD_2Cl_2) δ -105.82, -181.61.

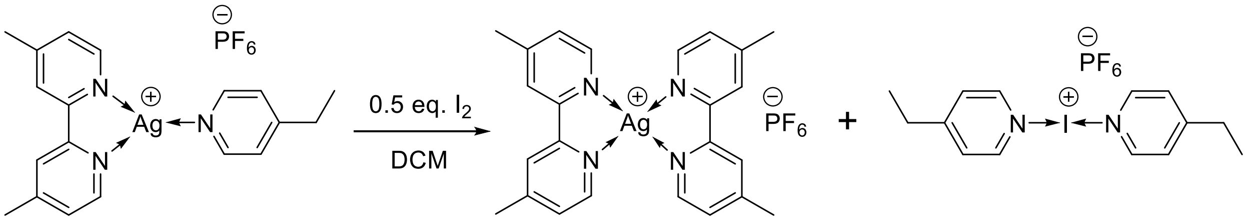

Pair of Complexes **10**: ^{1}H NMR (300 MHz, CD_2Cl_2) δ 8.58 (d, J = 6.6 Hz, 4H), 8.44 (d, J = 5.2 Hz, 4H), 8.06 (s, 4H), 7.39 (dd, J = 12.0, 5.5 Hz, 8H), 2.83 (q, J = 7.6 Hz, 4H), 2.56 (s, 12H), 1.30 (t, J = 7.6 Hz, 6H). ^{15}N NMR (500 MHz, CD_2Cl_2) δ -114.10, -181.74. Co-crystal **10** (from complex **4** + I_2): crystals suitable for single crystal X-ray diffraction were obtained from DCM vapor diffused with pentane.

Crystal data for **10**: CCDC-2062098, 5([$C_{24}H_{24}AgN_4$][PF_6])·[$C_{14}H_{18}IN_2$][PF_6], M = 3592.73, colorless plate, 0.03 x 0.07 x 0.20 mm^3, monoclinic, space group $P2_1$, a = 17.8170(7) Å, b = 20.3389(5) Å, c = 19.5885(6) Å, β = 97.333(3)°, V = 7040.4(4) Å^3, Z = 2, D_{calc} = 1.695 gcm^{-3}, F000 = 3596, μ = 8.77 mm^{-1}, T = 120.0(1) K, $θ_{max}$ = 76.0°, 27493 total reflections, 22558 with $I_o > 2σ(I_o)$, R_{int} = 0.046, 27493 data, 1860 parameters, 1 restraints, GooF = 1.01, 1.57 <dΔρ < -1.47 eÅ^{-3}, $R[F^2 > 2\sigma(F^2)]$ = 0.062, $wR(F^2)$ = 0.183.

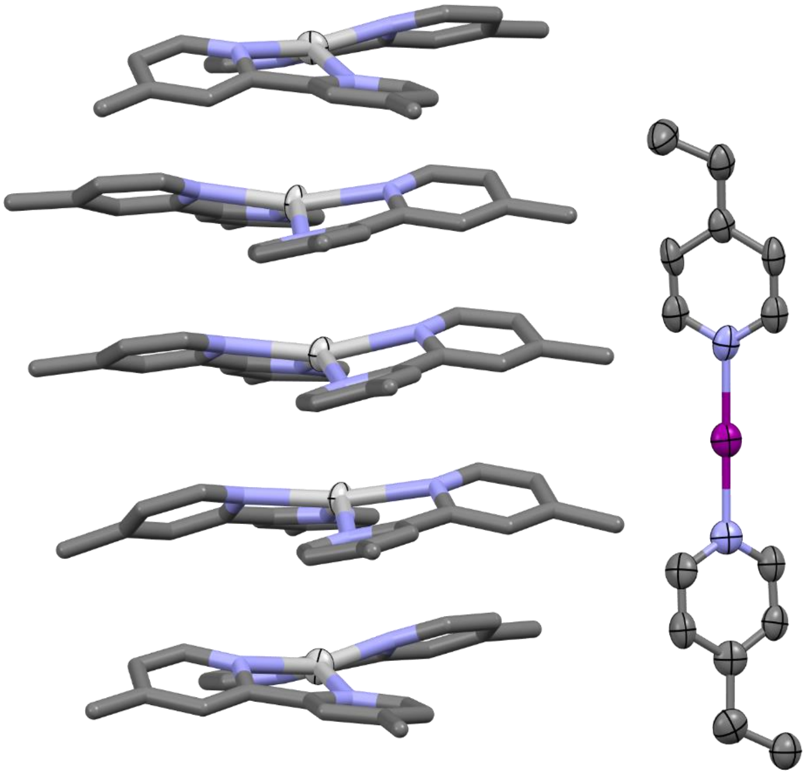

*Figure S6: The crystal structure of **8** (bpyMe$_2$ ligands simplified for clarity; thermal ellipsoids at 50% probability; PF$_6$ anions and hydrogen atoms omitted for clarity).*

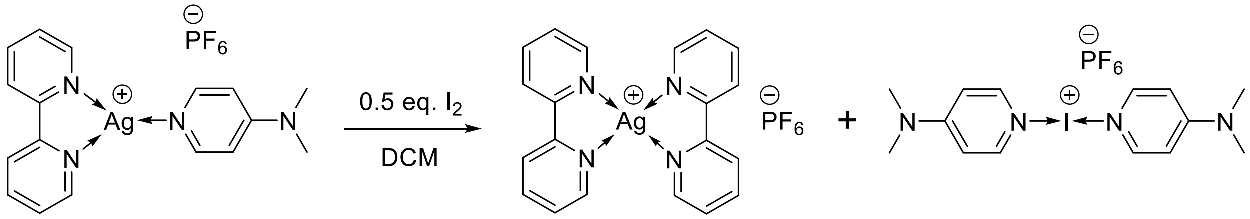

Pair of Complexes **11**: ^{1}H NMR (300 MHz, CD_2Cl_2) δ 8.64 (d, *J* = 4.9 Hz, 4H), 8.28 (d, *J* = 8.1 Hz, 4H), 8.13 – 8.03 (m, 8H), 7.58 (ddd, *J* = 7.3, 4.9, 0.8 Hz, 4H), 6.50 (d, *J* = 7.3 Hz, 4H), 3.11 (s, 12H). ^{15}N NMR (500 MHz, CD_2Cl_2) δ -105.81, -216.13.

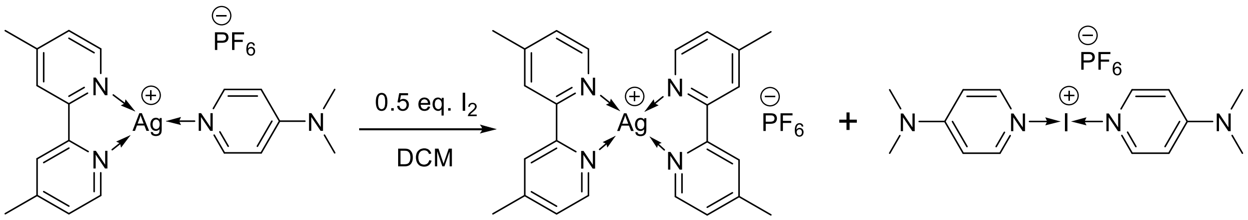

Pair of Complexes **12**: ^{1}H NMR (300 MHz, CD_2Cl_2) δ 8.44 (d, J = 5.1 Hz, 4H), 8.09 – 8.03 (m, 4H), 7.36 (d, J = 4.3 Hz, 4H), 6.50 (d, J = 7.3 Hz, 4H), 3.10 (s, 12H), 2.55 (s, 12H). ^{15}N NMR (500 MHz, CD_2Cl_2) δ -112.14, -216.01. Co-crystal **12** (from complex **6** + I_2): crystals suitable for single crystal X-ray diffraction were obtained from DCM vapor diffused with pentane.

Crystal data for **12**: CCDC-2062099, $[C_{24}H_{24}AgN_4][PF_6]\cdot[C_{14}H_{20}IN_4][PF_6]$, M = 1137.52, colorless plate, 0.02 x 0.08 x 0.10 mm^3, monoclinic, space group $C2/c$, a = 30.2860(5) Å, b = 7.20480(10) Å, c = 20.3464(4) Å, β = 101.206(2)°, V = 101.206(2) Å^3, Z = 4, D_{calc} = 1.735 gcm^{-3}, F000 = 2264, μ = 10.79 mm^{-1}, T = 120.0(1) K, θ_{max} = 76.3°, 4285 total reflections, 3981 with $I_o > 2\sigma(I_o)$, R_{int} = 0.029, 4285 data, 322 parameters, 117 restraints, GooF = 1.05, 1.53 <dΔρ < -0.70 eÅ^{-3}, $R[F^2 > 2\sigma(F^2)]$ = 0.032, $wR(F^2)$ = 0.083.

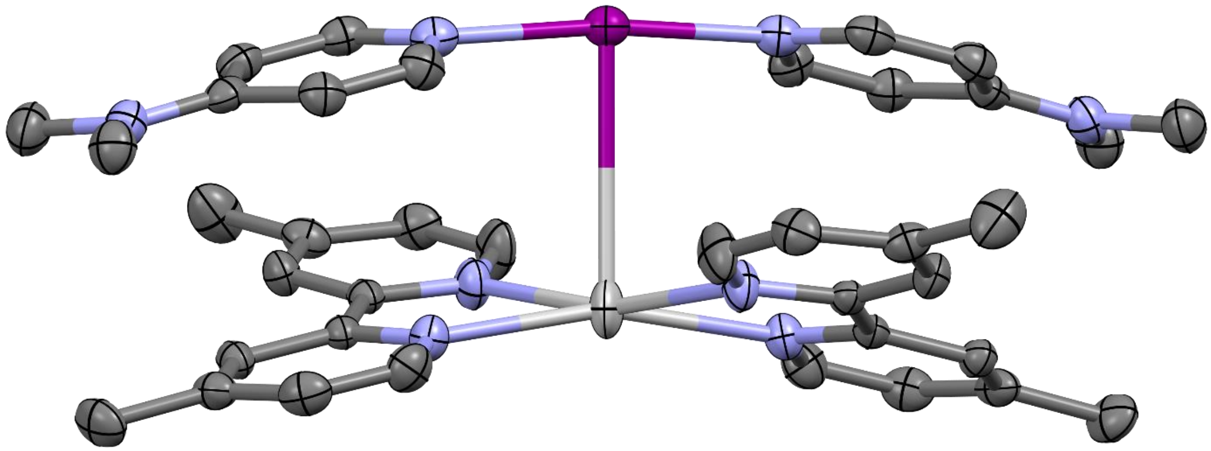

*Figure S7: The crystal structure of **9** ($I^+\cdots Ag^+$ = 3.4043 Å; thermal ellipsoids at 50% probability; PF_6 anions and hydrogen atoms omitted for clarity).*

Preparation of $[Ag(bpyMe_2)_2]PF_6$ (**13**): A DCM (2 mL) solution of $bpyMe_2$ (73.7 mg, 0.4 mmol) was added to a stirred MeCN (1 mL) solution of $AgPF_6$ (50.6 mg, 0.2 mmol) to give a pale yellow solution. Stirred for 30 minutes, then left to evaporate to dryness to give the product as a yellow solid in quantitative yield. Crystals suitable for single crystal X-ray diffraction were obtained from partial evaporation of a DCM/MeCN (3:1 ratio) solution (**13_1**) and from DCM vapor diffused with diisopropylether (**13_2**). ^{1}H NMR (300 MHz, CD_2Cl_2) δ 8.43 (d, *J* = 5.2 Hz, 4H), 8.05 (s, 4H), 7.37 (d, *J* = 4.5 Hz, 4H), 2.55 (s, 12H).). ^{15}N NMR (500 MHz, CD_2Cl_2) δ -114.35. Analysis Found: C, 46.52; H, 3.96: N, 9.07%. Calculated for $C_{24}H_{24}AgF_6N_4P$: C, 46.40; H, 3.89: N, 9.02%. Crystal data for **13_1**: CCDC-2062100, $[C_{24}H_{24}AgN_4][PF_6]$, M = 621.31, colorless plate, 0.03 x 0.13 x 0.30 mm^3, triclinic, space group *P*-1 (No. 2), a = 8.1664(3) Å, b = 11.3408(8) Å, c = 13.8483(8) Å, α = 101.019(5)°, β = 91.176(4)°, γ = 105.166(5)°, V = 1211.68(12) Å^3, Z = 2, D_{calc} = 1.703 gcm^{-3}, F000 = 624, μ = 7.91 mm^{-1}, T = 120.0(1) K, θ_{max} = 76.6°, 4845 total reflections, 4531 with $I_o > 2\sigma(I_o)$, R_{int} = 0.020, 4845 data, 329 parameters, no restraints, GooF = 1.04, 0.35 <dΔρ < -0.60 eÅ^{-3}, $R[F^2 > 2\sigma(F^2)]$ = 0.026, $wR(F^2)$ = 0.068.

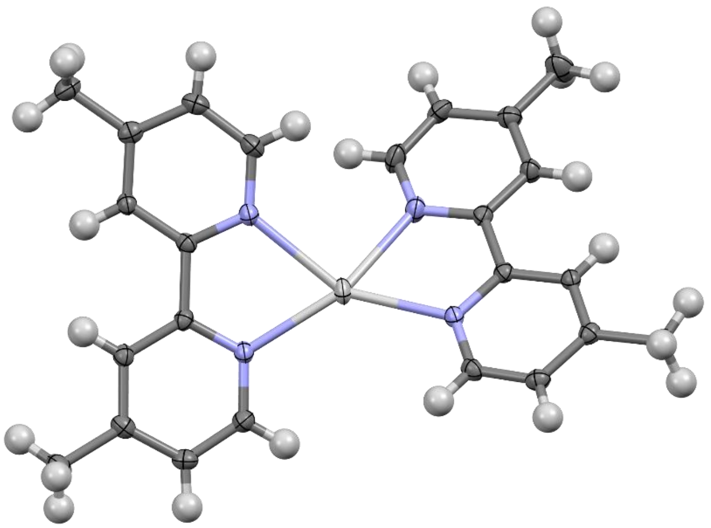

*Figure S8: The crystal structure of **13_1** (thermal ellipsoids at 50% probability; PF_6 anion omitted for clarity).*

Crystal data for **13_2**: CCDC-2062101, ($[C_{24}H_{24}AgN_4][PF_6])_2$, M = 1242.62, colorless plate, 0.06 x 0.12 x 0.16 mm^3, monoclinic, space group $P2_1/c$, a = 19.9049(7) Å, b = 20.1225(8) Å, c = 14.3054(3) Å, β = 107.474(2)°, V = 5465.4(3) Å^3, Z = 4, D_{calc} = 1.510 gcm^{-3}, F000 = 2496, μ = 0.86 mm^{-1}, T = 170(1) K, θ_{max} = 27.9°, 9630 total reflections, 4846 with $I_o > 2\sigma(I_o)$, R_{int} = 0.094, 9630 data, 713 parameters, 459 restraints, GooF = 1.06, 0.84 <dΔρ < -0.69 eÅ^{-3}, $R[F^2 > 2\sigma(F^2)]$ = 0.099, $wR(F^2)$ = 0.283. Unknown solvates were also found to be present in this structure, however, they could not be identified due to being heavily disordered. No sensible disordered model could be formulated for the unknown solvates which would match the observed electron density, so the computer program SQUEEZE within PLATON was used to account for the electron density in this region of the unit cell.[9] The program identified solvent accessible voids totalling 646.0 Å^3 and 401.1 electrons per unit cell were recovered.

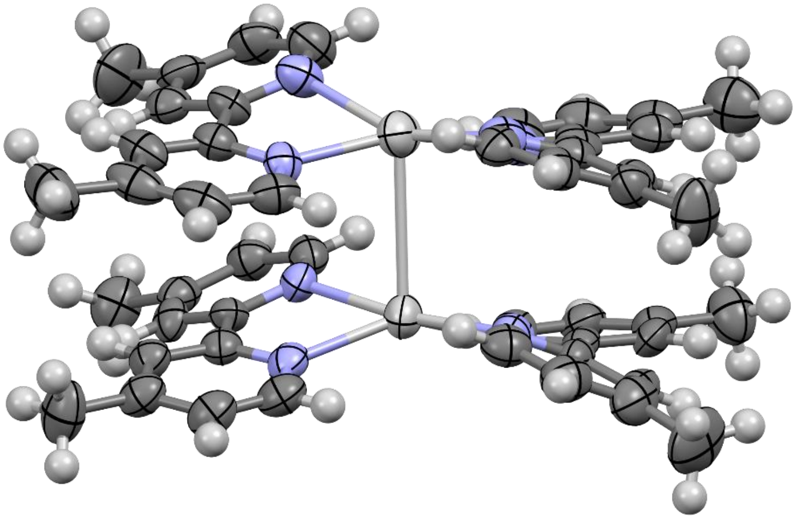

*Figure S9: The crystal structure of **13_2**, which was observed as a dimer (Ag^+---Ag^+ = 3.165(1) Å; thermal ellipsoids at 50% probability; PF_6 anions omitted for clarity).*

Comparison Tables of ^{15}N NMR Resonances for Ag^{+}–N and I^{+}–N

**Complex**	**^{15}N (ppm)**	**Complex**	**^{15}N (ppm)**
$[Ag(bpy)(mtz)]PF_6$ (**1**)			
N(bpy)	-111.49(2)	$[Ag(bpy)_2]PF_6$	-106.48(5)
N(mtz)	-141.36(2)		
$[Ag(bpyMe_2)(mtz)]PF_6$ (**2**)			
$N(bpyMe_2)$	-119.22(2)	$[Ag(bpyMe_2)_2]PF_6$ (**13**)	-114.35(6)
N(mtz)	-141.63(3)		
$[Ag(bpy)(4\text{-}Etpy)]PF_6$ (**3**)			
N(bpy)	-110.10(5)	$[I(mtz)_2]PF_6$	-142.45(2)
N(4-Etpy)	-132.55(4)		
$[Ag(bpyMe_2)(4\text{-}Etpy)]PF_6$ (**4**)			
$N(bpyMe_2)$	-117.90(5)	$[I(4\text{-}Etpy)_2]PF_6$	-181.61(2)
N(4-Etpy)	-130.76(5)		
$[Ag(bpy)(4\text{-}DMAP)]PF_6$ (**5**)			
N(bpy)	-108.73(3)	$[I(4\text{-}DMAP)_2]PF_6$	-216.09(4)
N(4-DMAP)	-169.62(2)		
$[Ag(bpyMe_2)(4\text{-}DMAP)]PF_6$ (**6**)			
$N(bpyMe_2)$	-116.49(2)		
N(4-DMAP)	-169.95(2)		

Table S1: The ^{15}N NMR resonances (determined by ^{1}H-^{15}N HMBC experiments) of the coordinating nitrogen atoms for all pure compounds discussed in this work (with the error of the measurement given in brackets).

**Pair of Complexes**	^{15}**N (ppm)**	**Pair of Complexes**	^{15}**N (ppm)**
**7** (**1** + I_2)		**8** (**2** + I_2)	
$[Ag(bpy)_2]PF_6$	-109.73(1)	$[Ag(bpyMe_2)_2]PF_6$	(not observed)
$[I(mtz)_2]PF_6$	-142.35(1)	$[I(mtz)_2]PF_6$	-142.21(2)
**9** (**3** + I_2)		**10** (**4** + I_2)	
$[Ag(bpy)_2]PF_6$	-105.82(4)	$[Ag(bpyMe_2)_2]PF_6$	-114.10(5)
$[I(4\text{-}Etpy)_2]PF_6$	-181.61(1)	$[I(4\text{-}Etpy)_2]PF_6$	-181.74(4)
**11** (**5** + I_2)		**12** (**6** + I_2)	
$[Ag(bpy)_2]PF_6$	-105.81(4)	$[Ag(bpyMe_2)_2]PF_6$	-112.14(8)
$[I(4\text{-}DMAP)_2]PF_6$	-216.13(2)	$[I(4\text{-}DMAP)_2]PF_6$	-216.01(4)

Table S2: The ^{15}N NMR resonances (determined by ^{1}H-^{15}N HMBC experiments) of the coordinating nitrogen atoms for all the pairs of complexes discussed in this work (with the error of the measurement given in brackets). Resonances that have significantly shifted from those of their parent pure complexes (see Table S1) are highlighted in red.

NMR Spectra

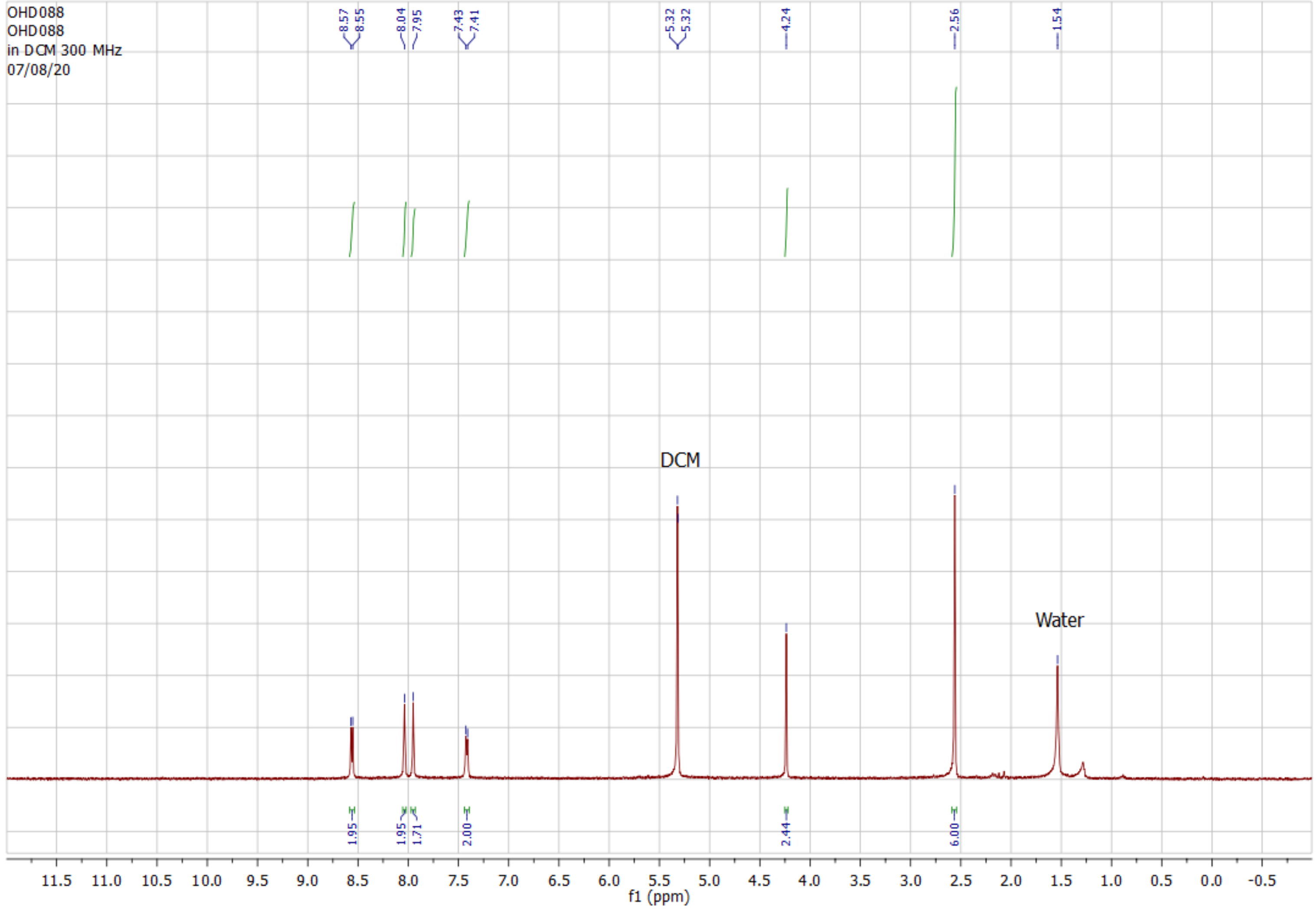

*Figure S10: The ^{1}H NMR spectrum of complex **2** in CD_2Cl_2.*

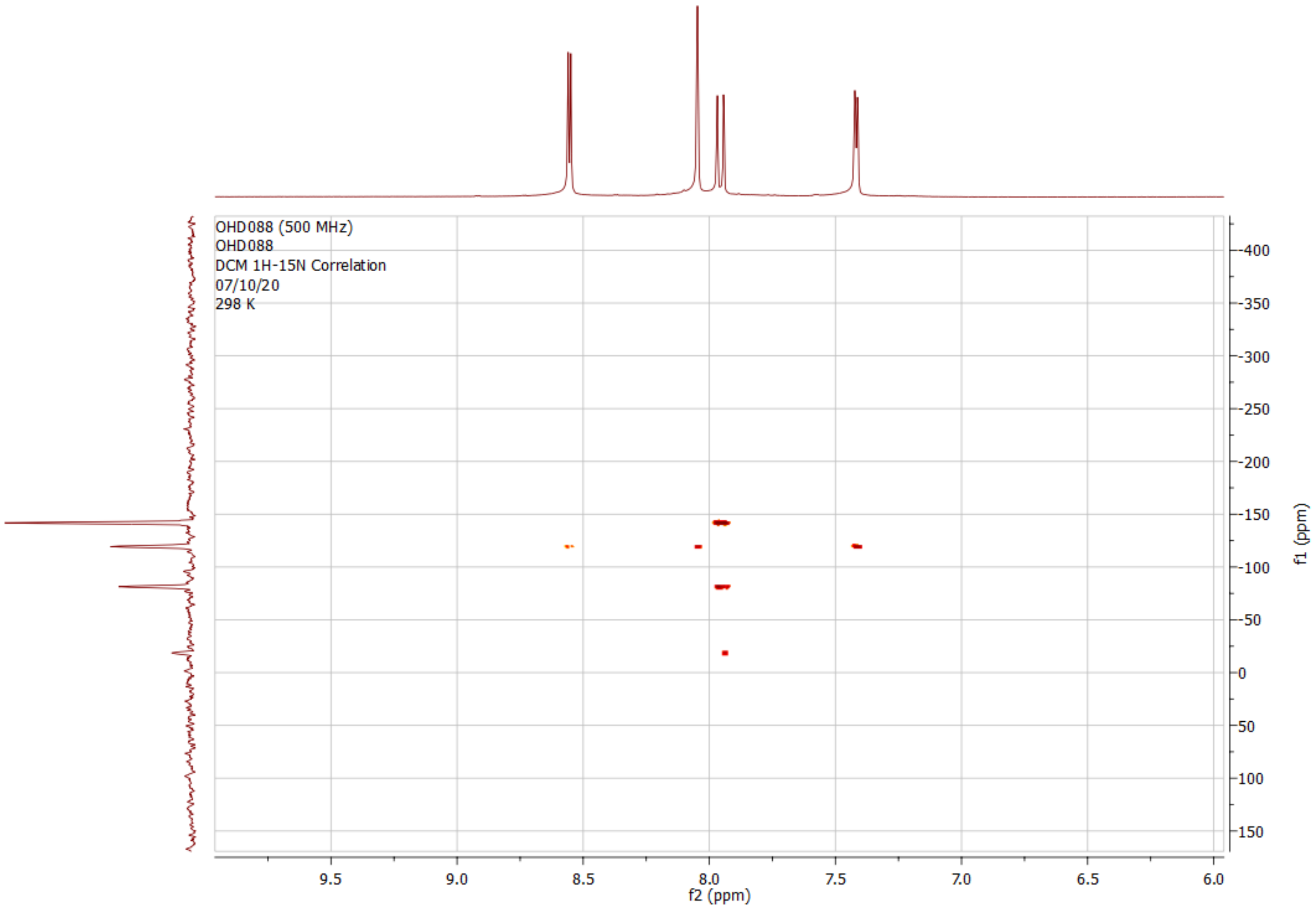

Figure S11: The ^{1}H-^{15}N *NMR HMBC spectrum of complex* ***2*** *in* CD_2Cl_2.

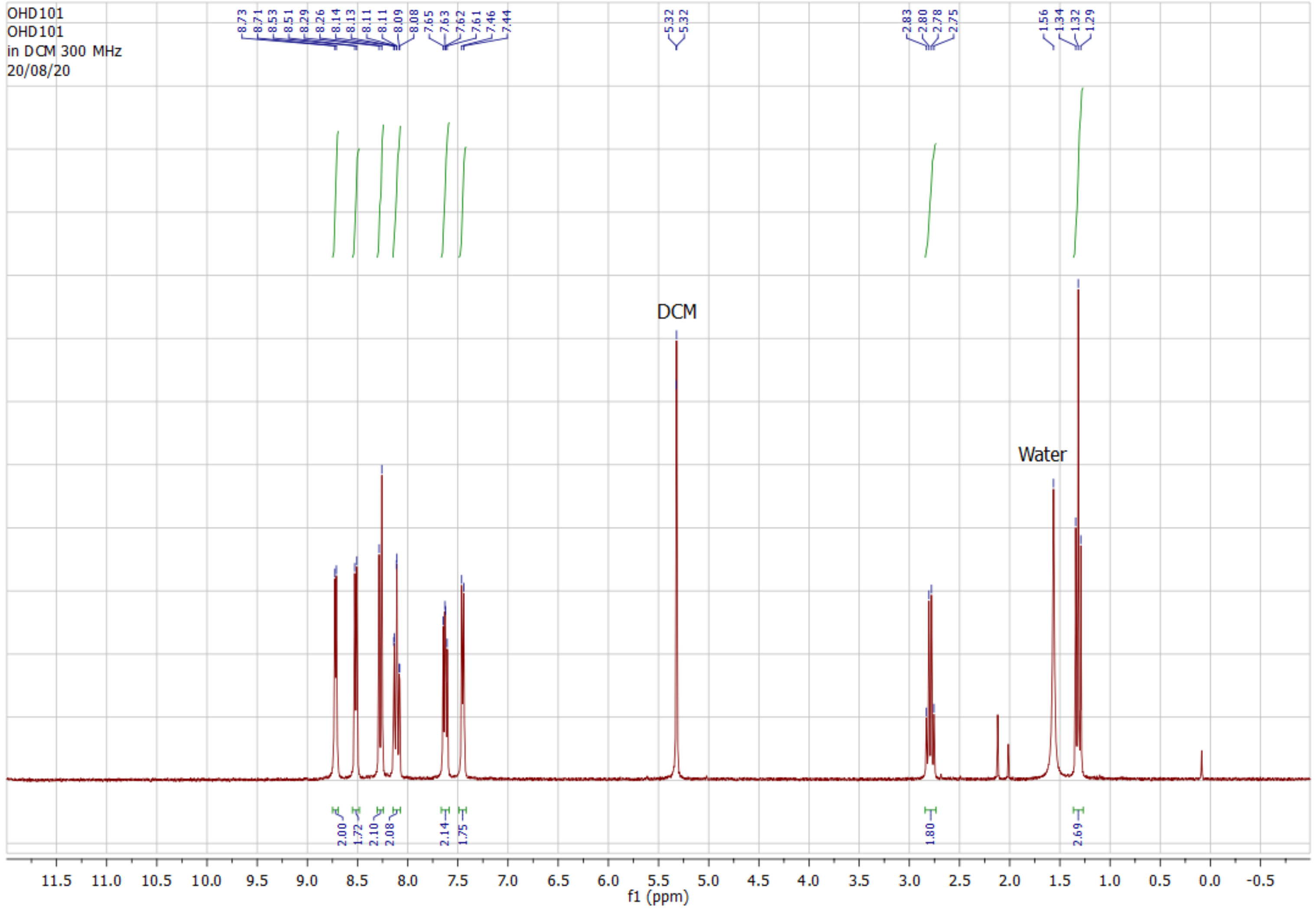

*Figure S12: The ^{1}H NMR spectrum of complex **3** in CD_2Cl_2.*

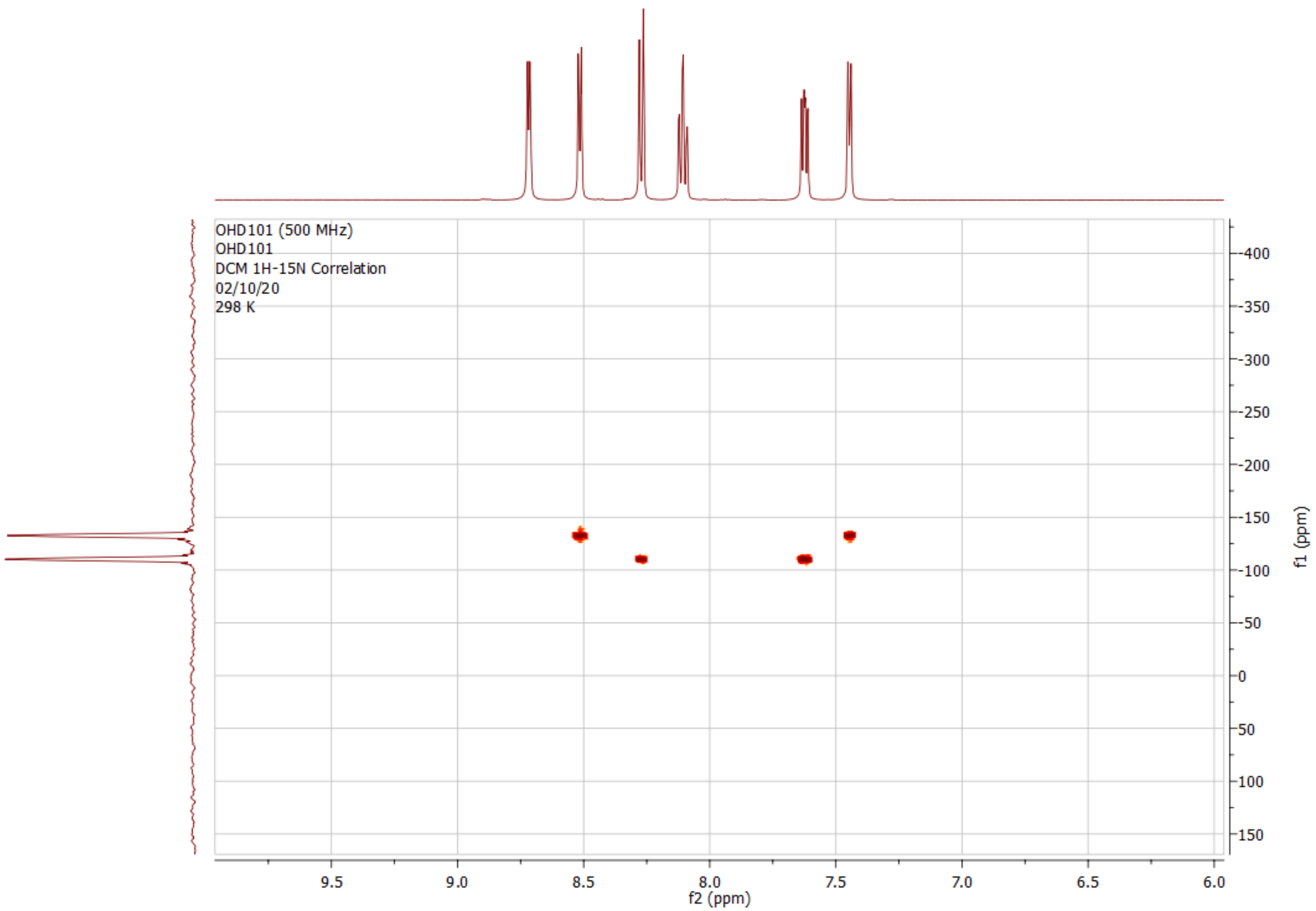

*Figure S13: The ^{1}H-^{15}N NMR HMBC spectrum of complex **3** in CD_2Cl_2.*

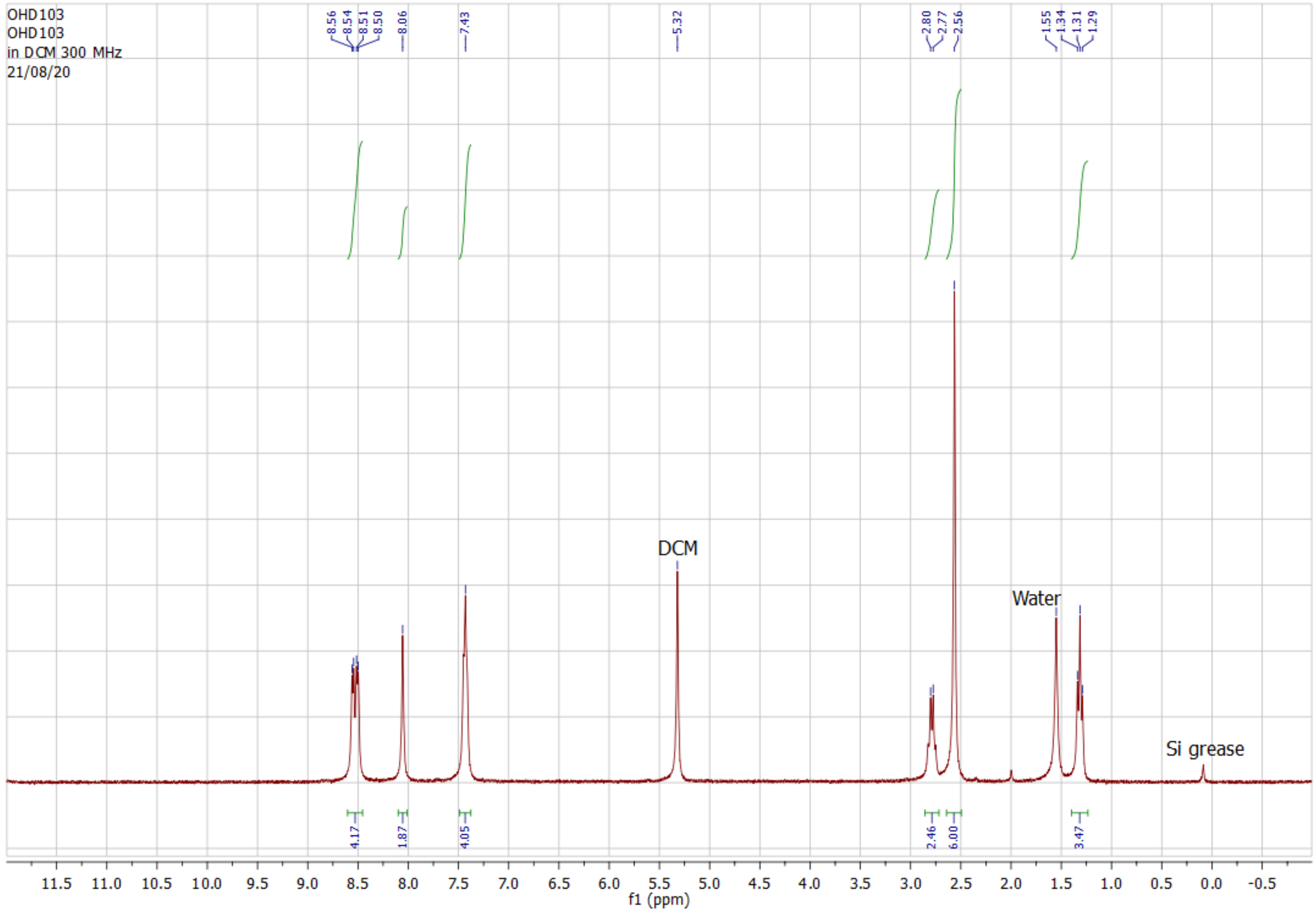

*Figure S14: The ^{1}H NMR spectrum of complex **4** in CD_2Cl_2.*

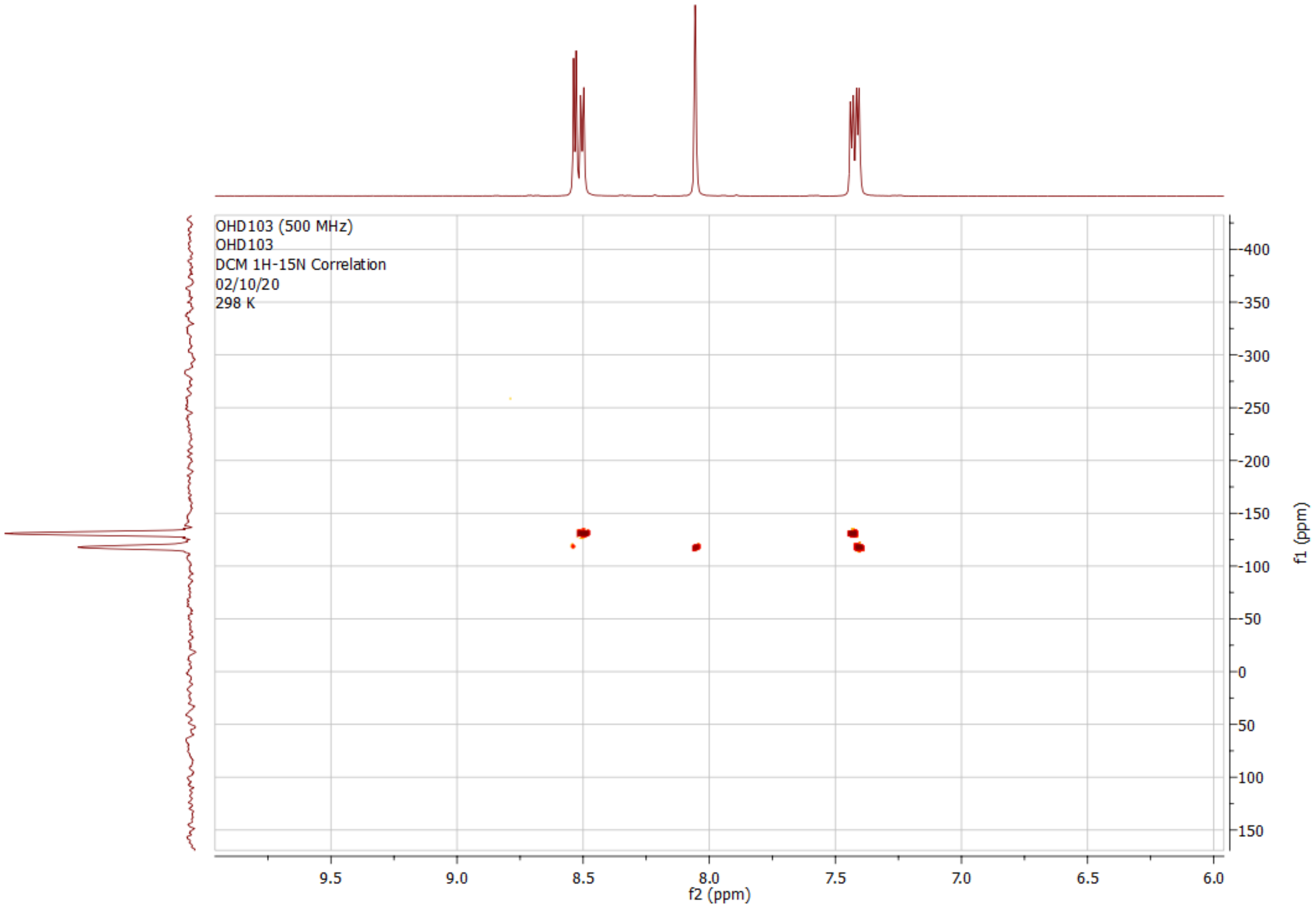

*Figure S15: The ^{1}H-^{15}N NMR HMBC spectrum of complex **4** in CD_2Cl_2.*

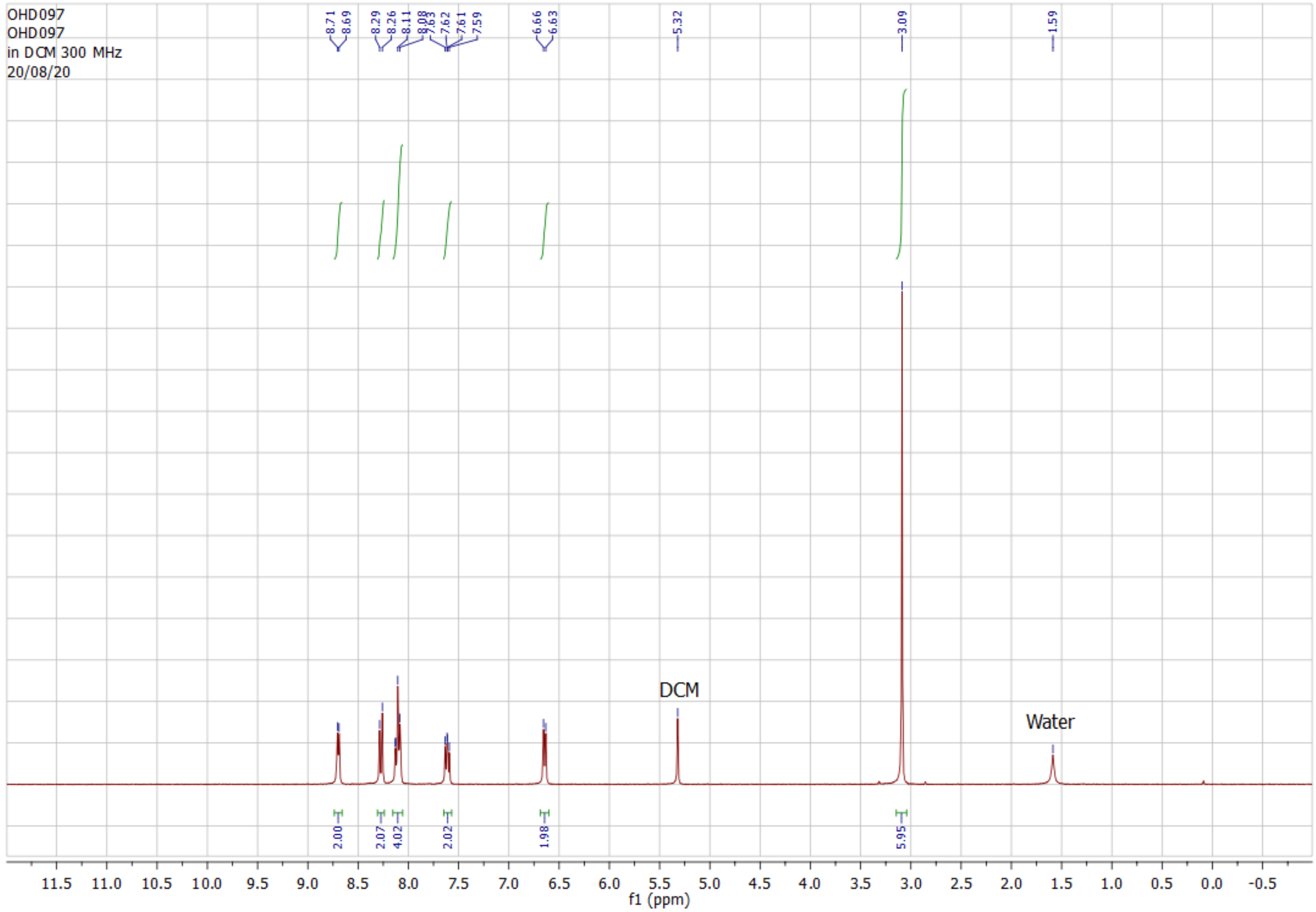

*Figure S16: The ^{1}H NMR spectrum of complex **5** in CD_2Cl_2.*

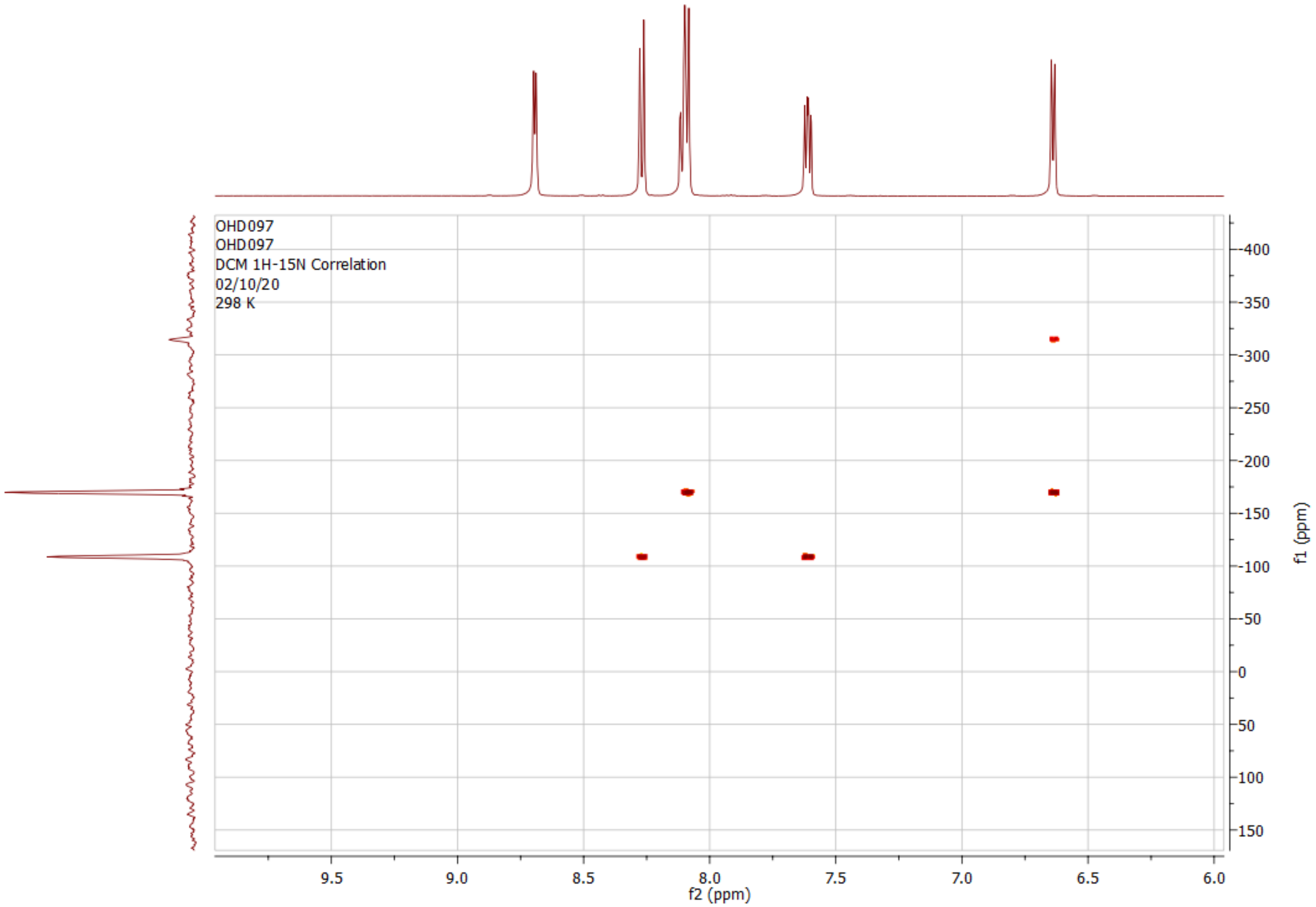

*Figure S17: The ^{1}H-^{15}N NMR HMBC spectrum of complex **5** in CD_2Cl_2.*

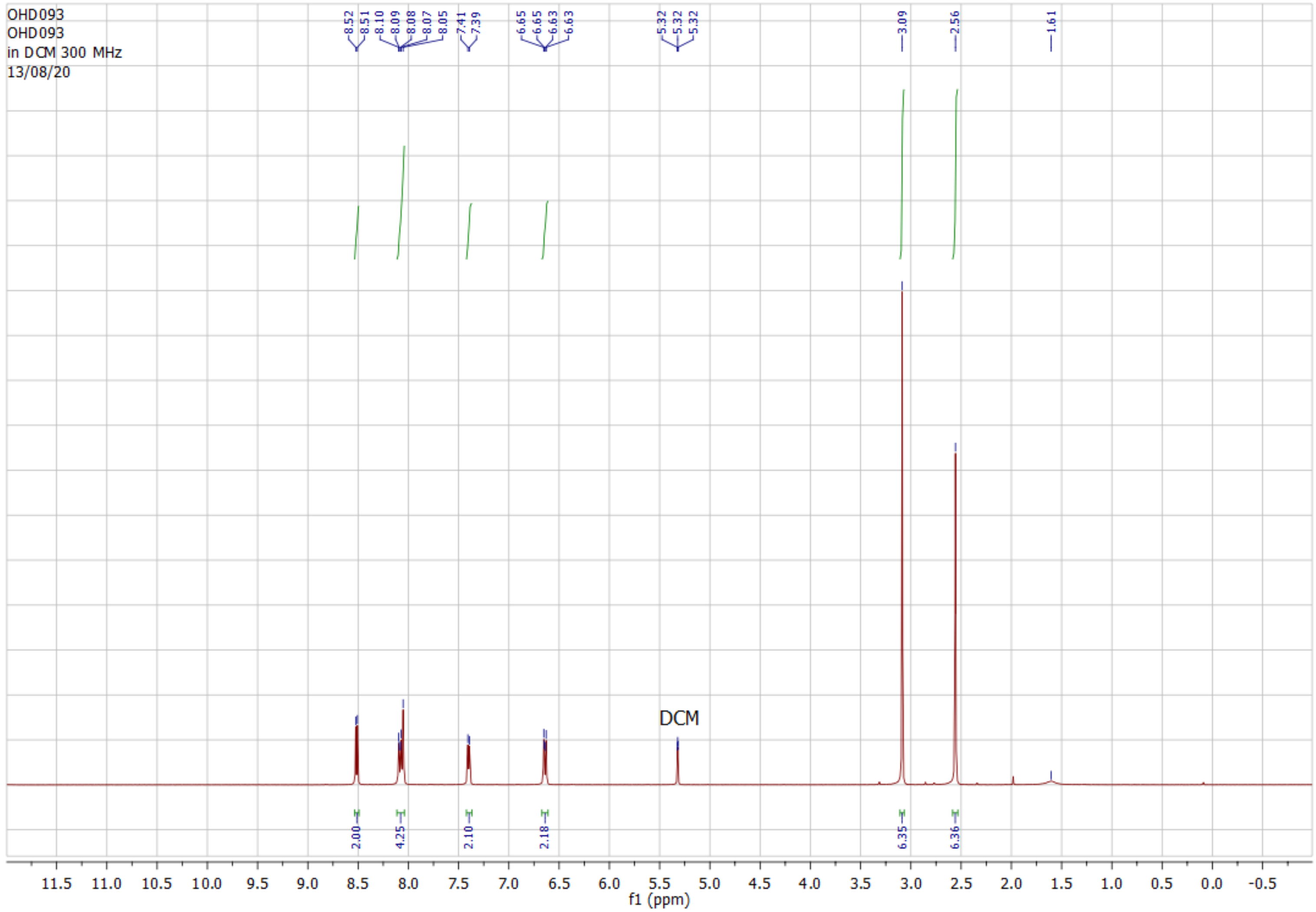

*Figure S18: The ^{1}H NMR spectrum of complex **6** in CD_2Cl_2.*

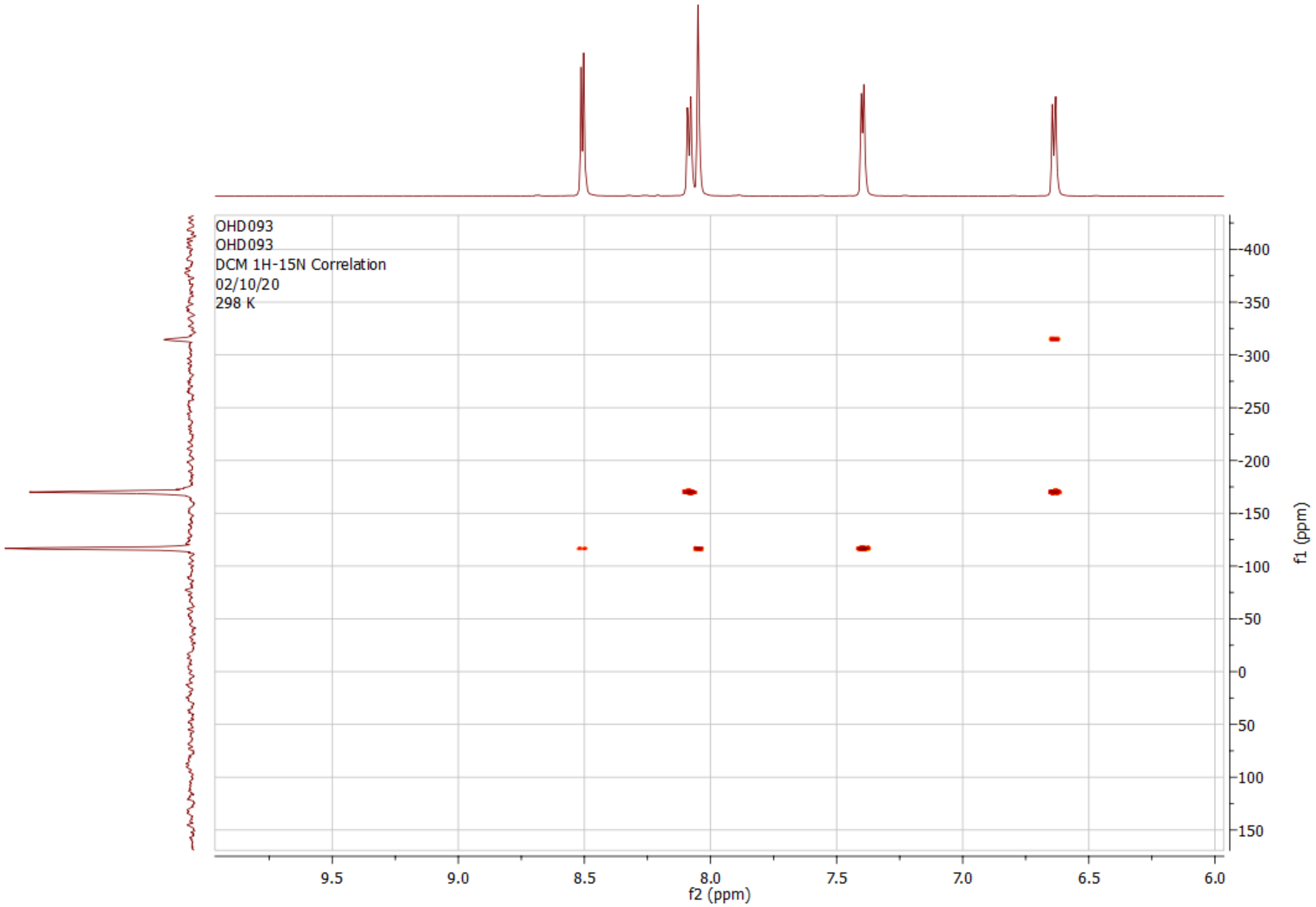

*Figure S19: The ^{1}H-^{15}N NMR HMBC spectrum of complex **6** in CD_2Cl_2.*

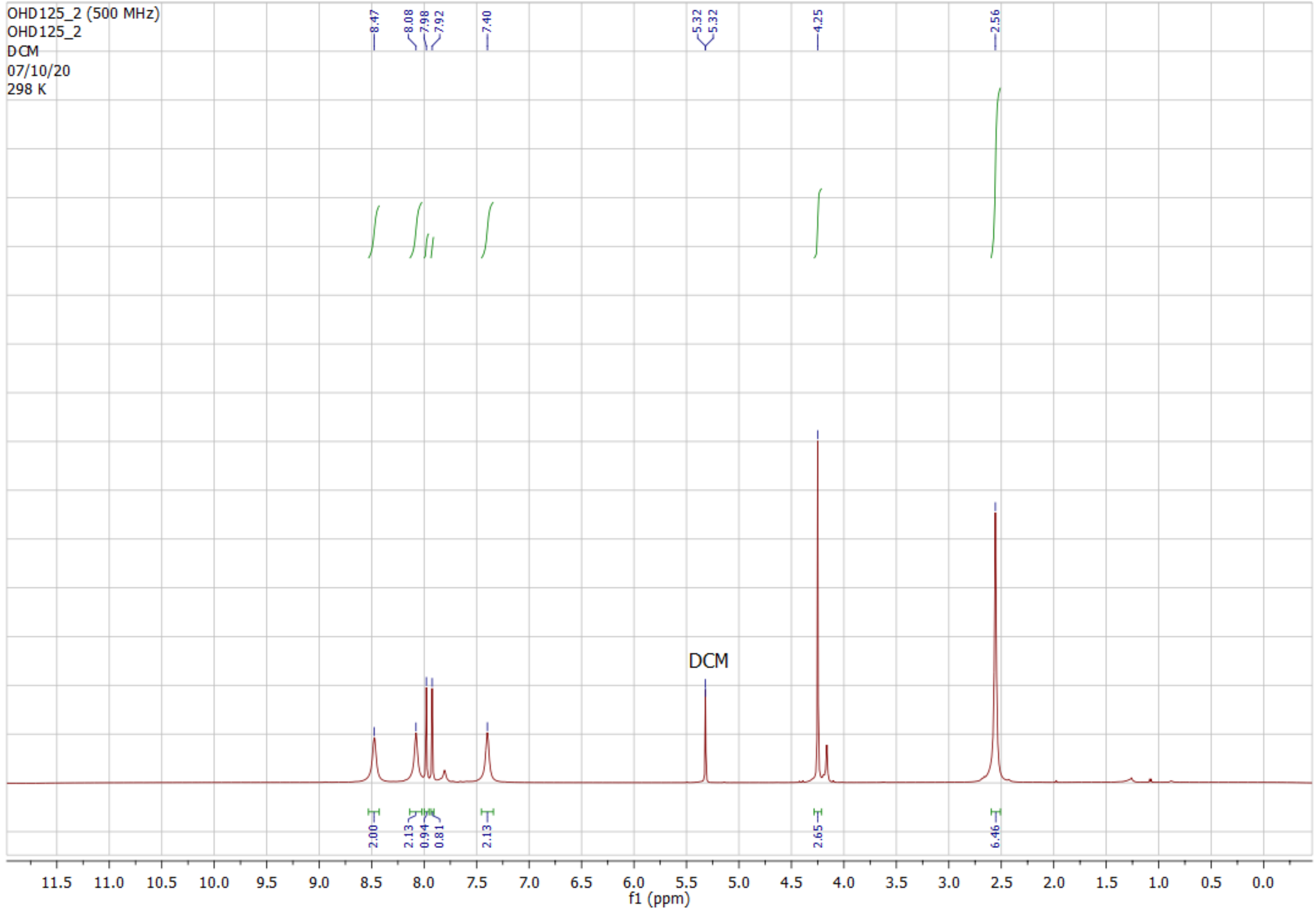

*Figure S20: The ^{1}H NMR spectrum of the pair of complexes **8** in CD_2Cl_2.*

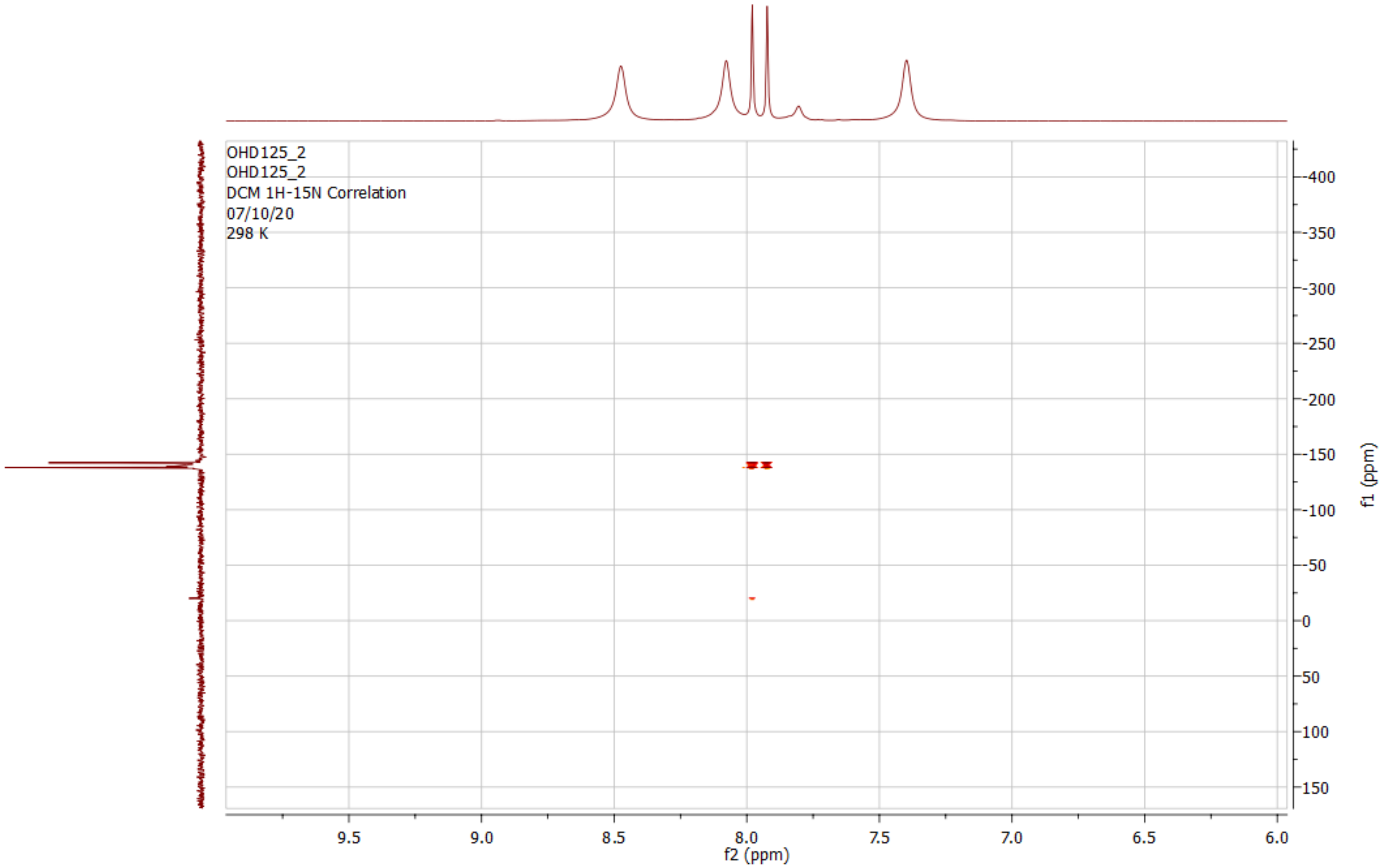

*Figure S21: The ^{1}H-^{15}N NMR HMBC spectrum of the pair of complexes **8** in CD_2Cl_2.*

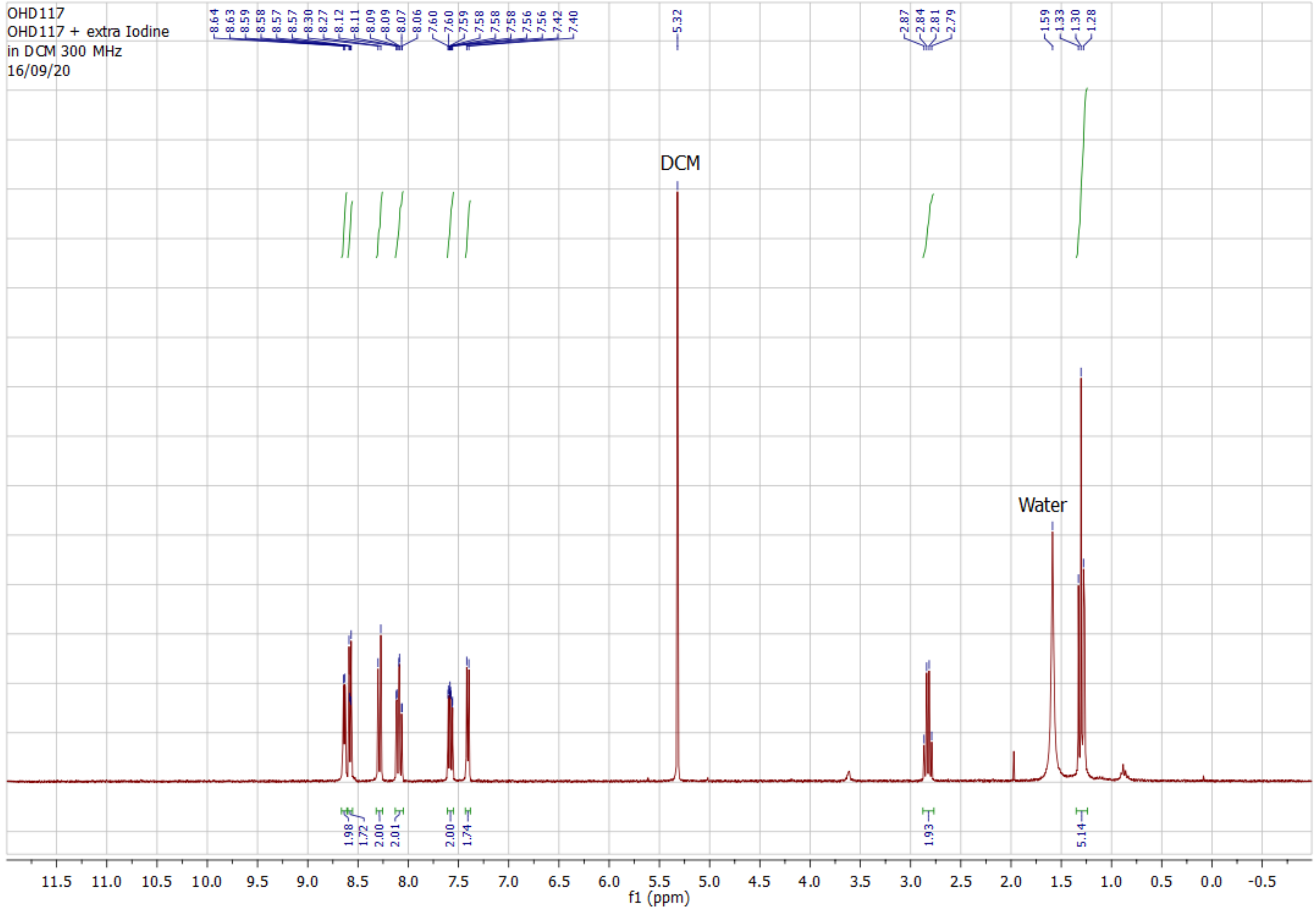

Figure S22: The ^{1}H NMR spectrum of the pair of complexes ***9*** *in CD_2Cl_2.*

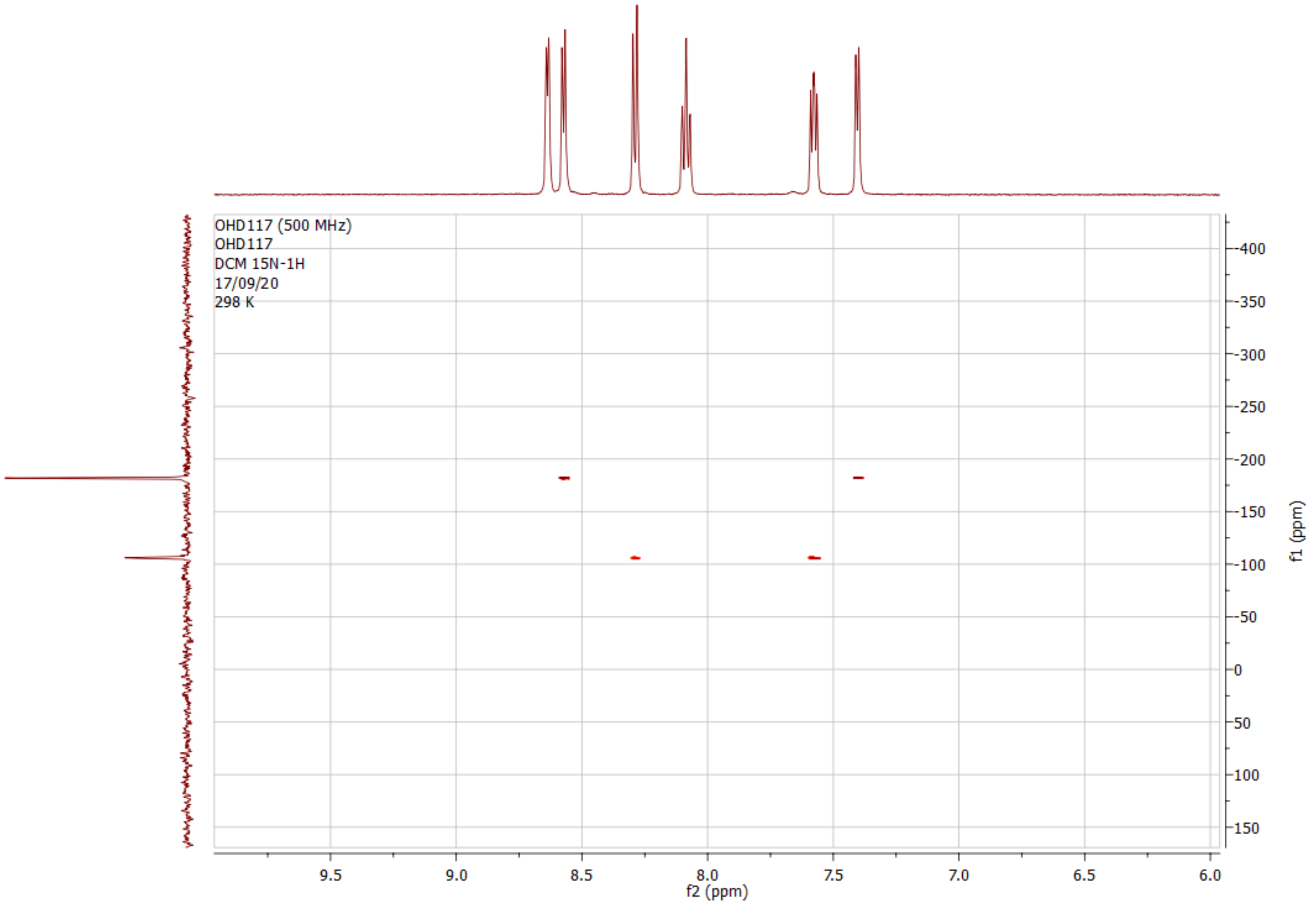

Figure S23: The ^{1}H-^{15}N NMR HMBC spectrum of the pair of complexes ***9*** *in CD_2Cl_2.*

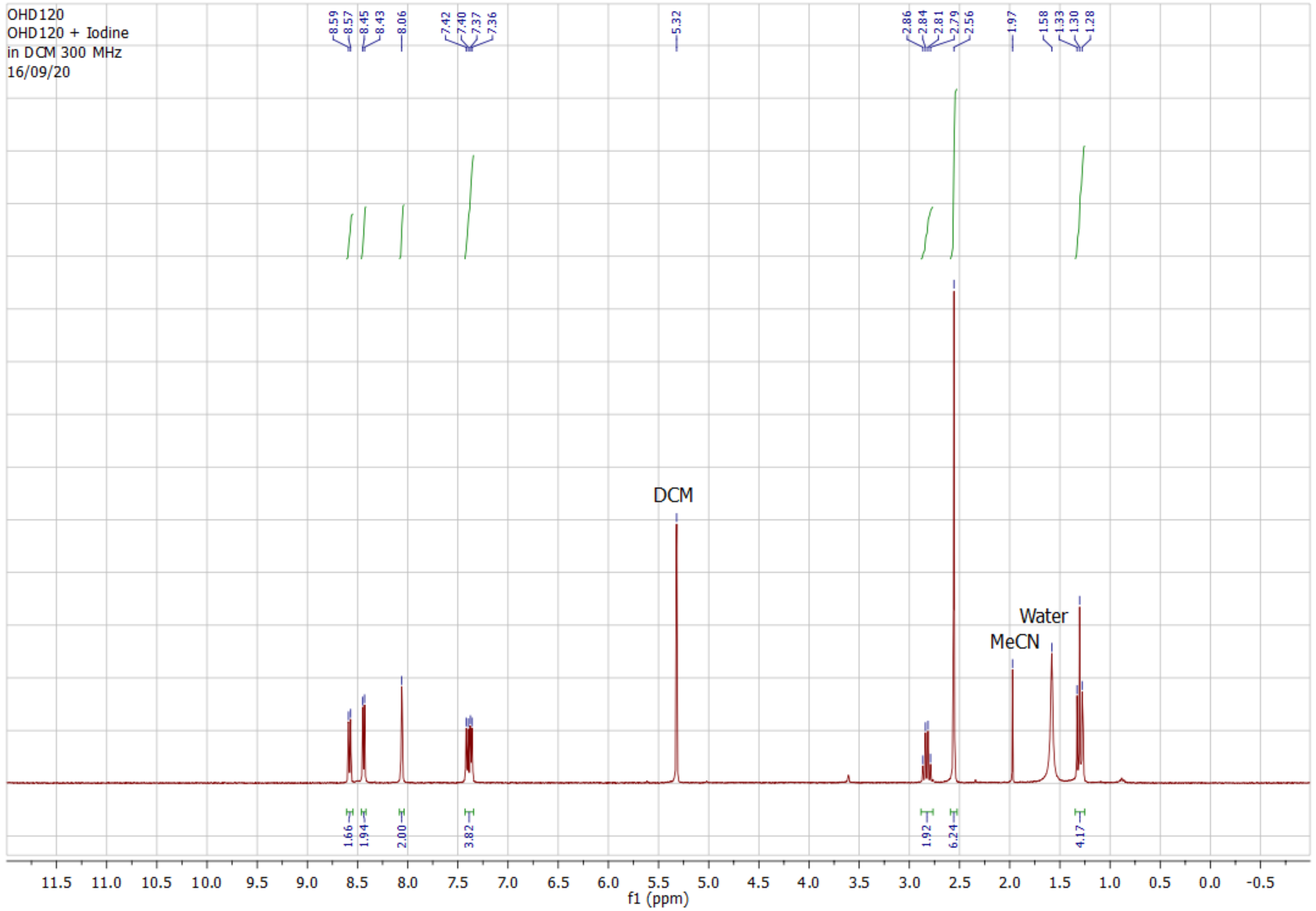

*Figure S24: The ^{1}H NMR spectrum of the pair of complexes **10** in CD_2Cl_2.*

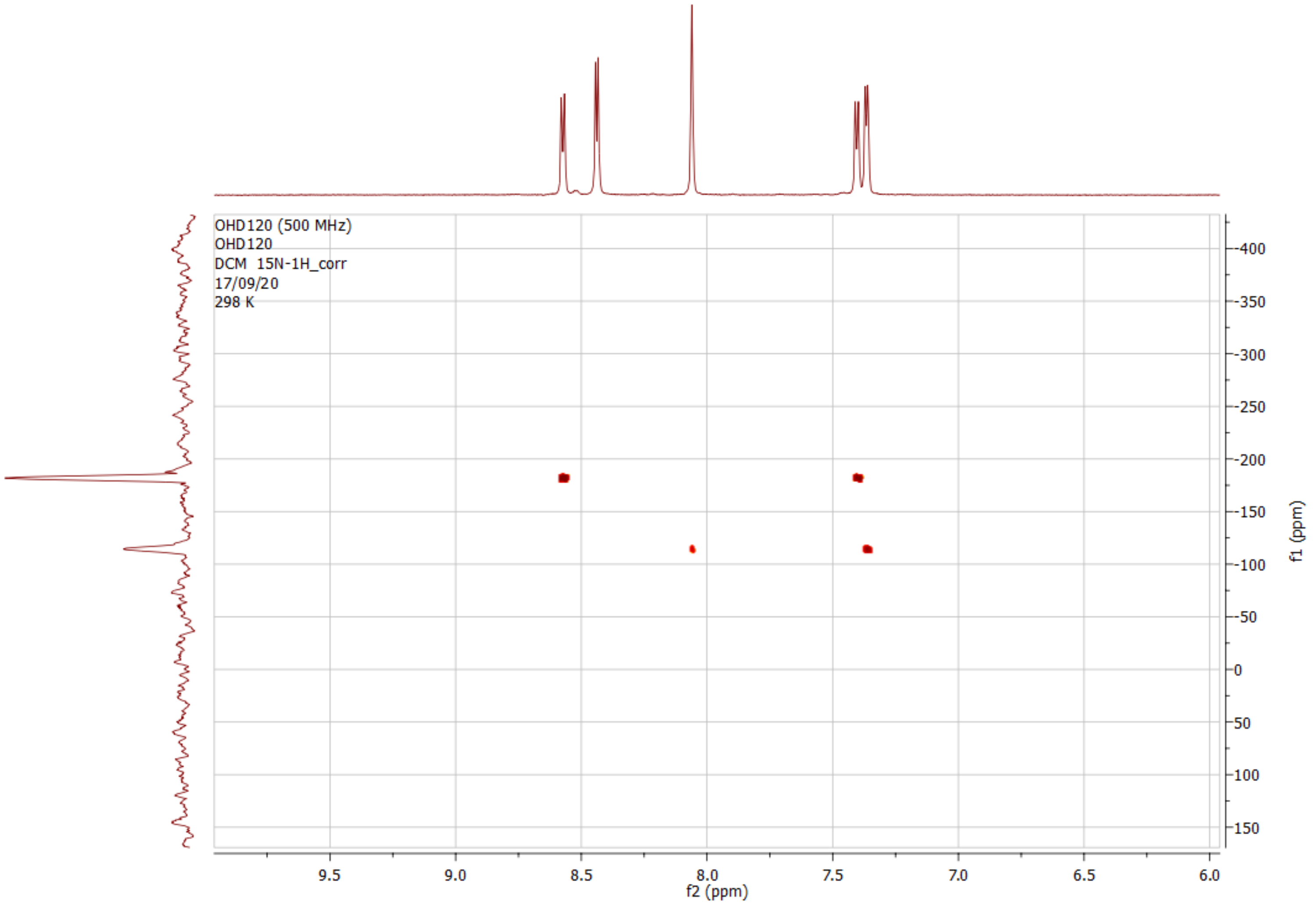

*Figure S25: The ^{1}H-^{15}N NMR HMBC spectrum of the pair of complexes **10** in CD_2Cl_2.*

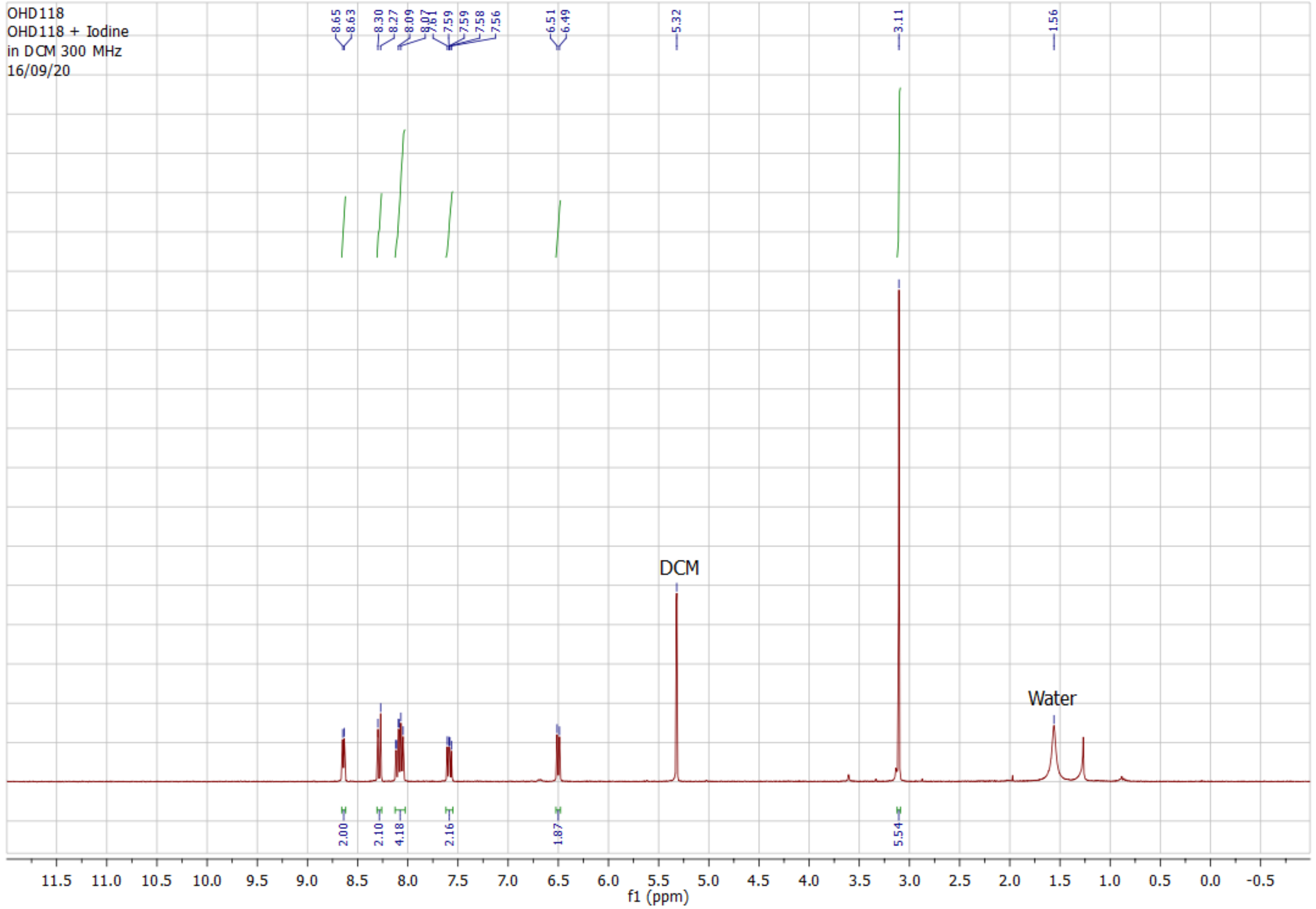

*Figure S26: The ^{1}H NMR spectrum of the pair of complexes **11** in CD_2Cl_2.*

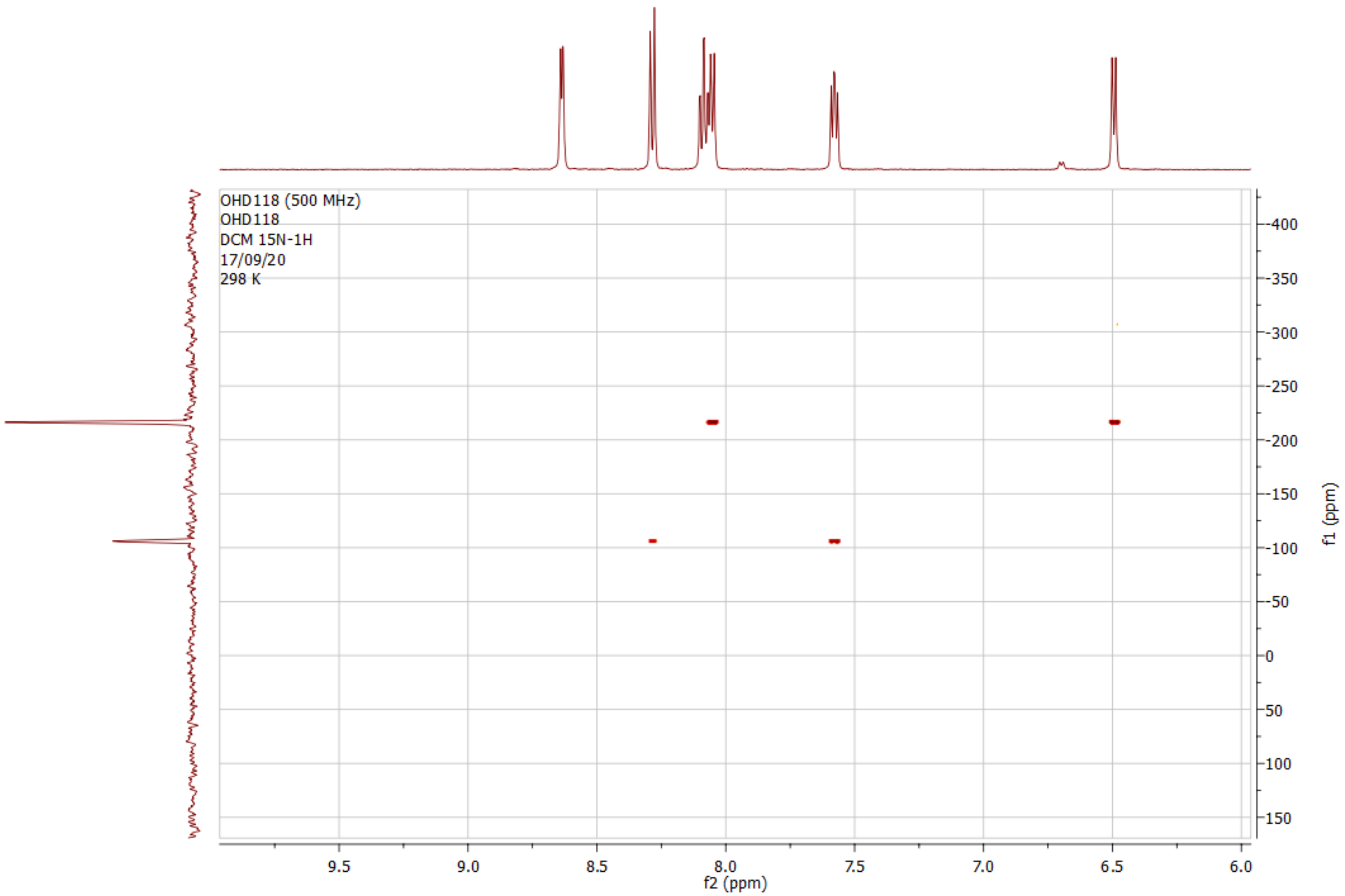

*Figure S27: The ^{1}H-^{15}N NMR HMBC spectrum of the pair of complexes **11** in CD_2Cl_2.*

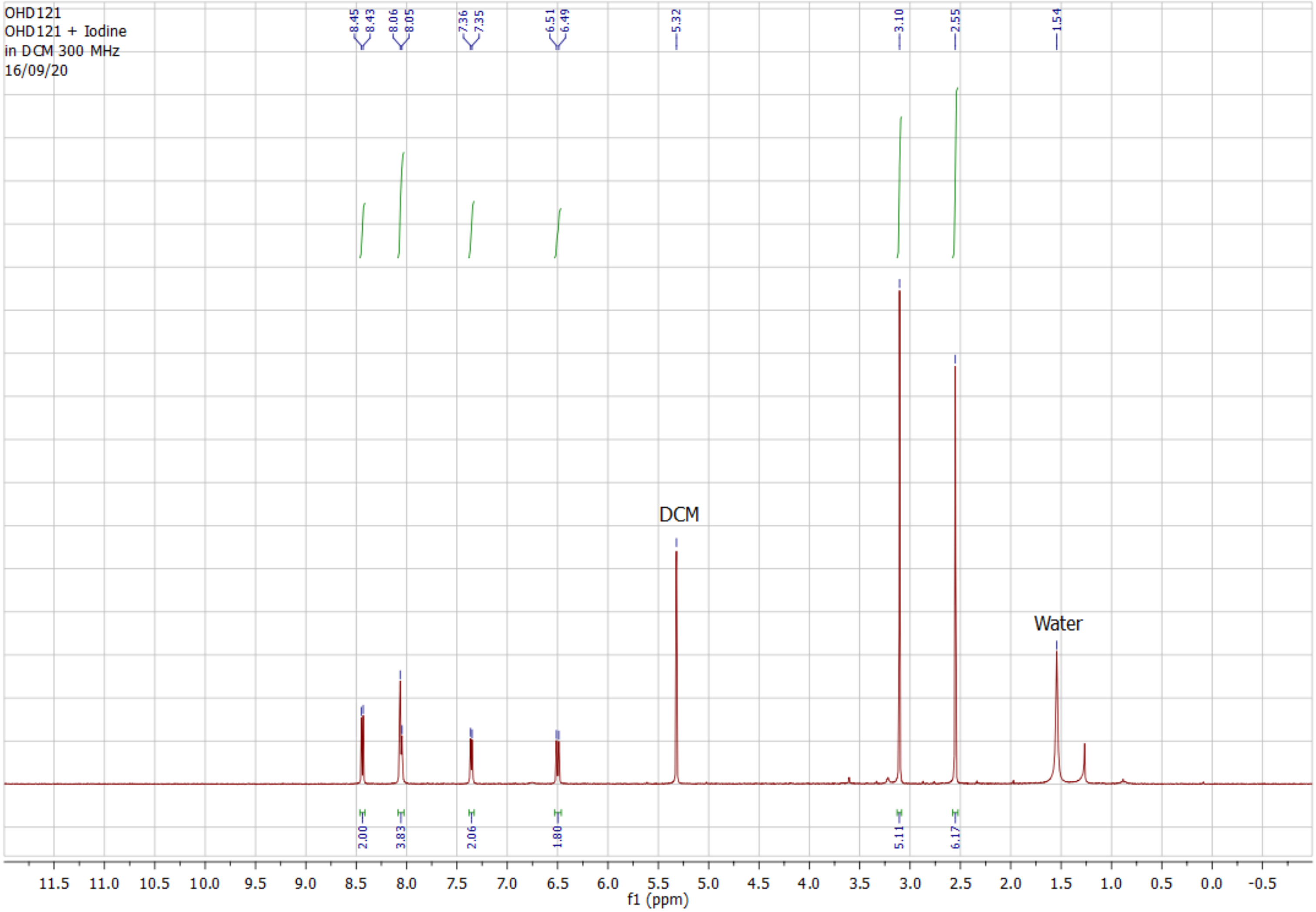

*Figure S28: The ^{1}H NMR spectrum of the pair of complexes **12** in CD_2Cl_2.*

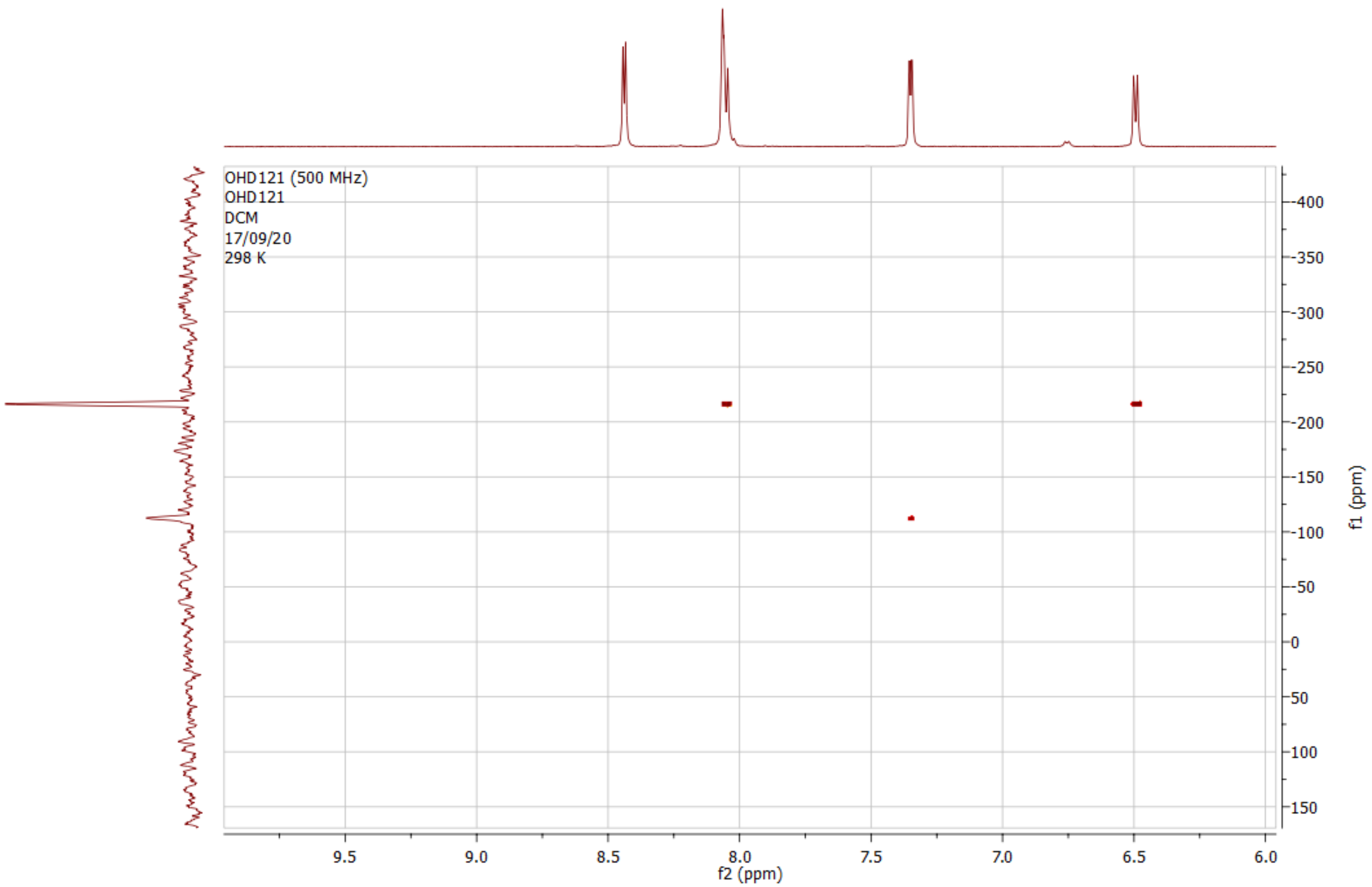

Figure S29: The ^{1}H-^{15}N NMR HMBC spectrum of the pair of complexes ***12*** *in* CD_2Cl_2.

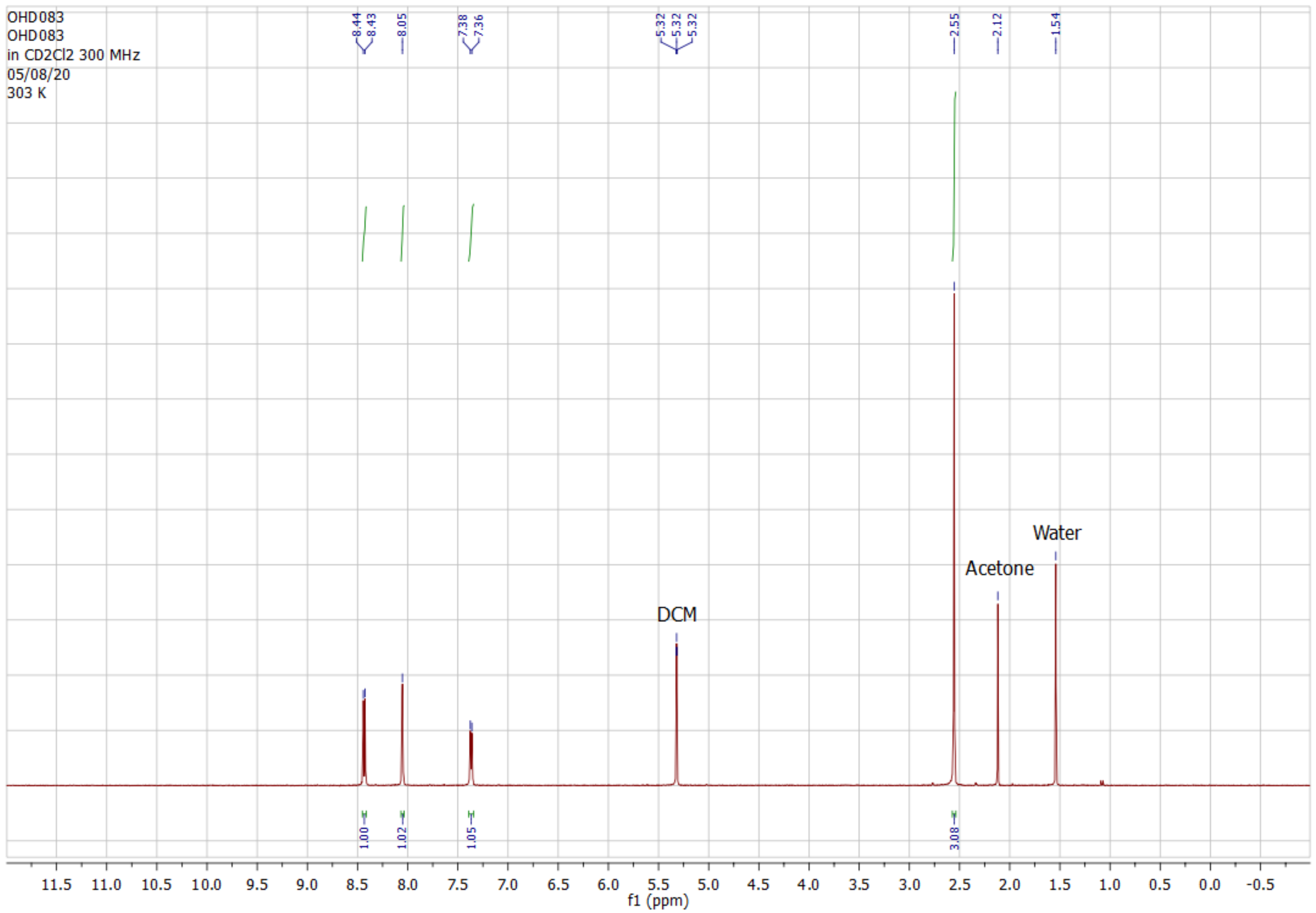

*Figure S30: The ^{1}H NMR spectrum of complex **13** in CD_2Cl_2.*

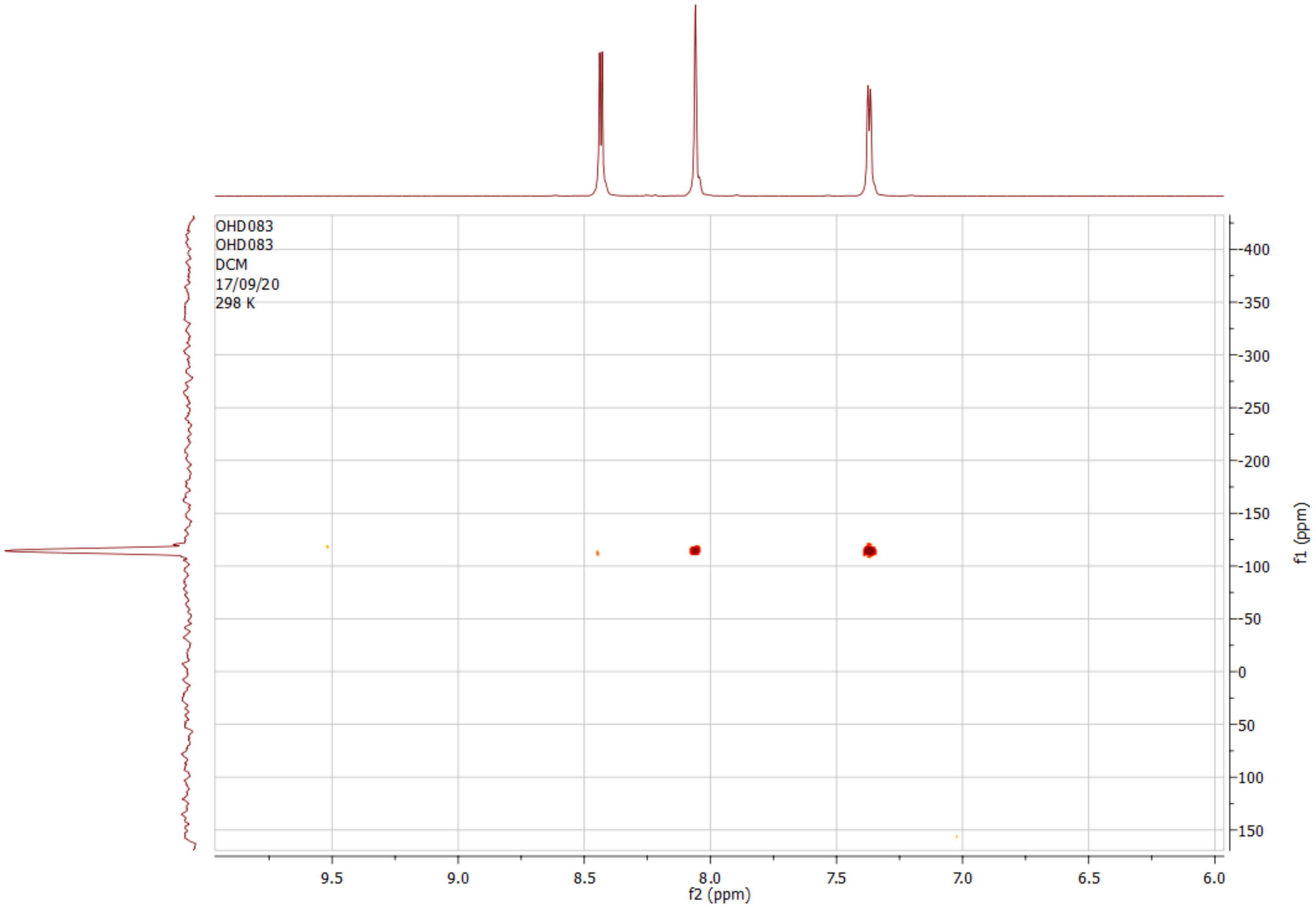

Figure S31: The ^{1}H-^{15}N NMR HMBC spectrum of complex ***13*** *in CD_2Cl_2.*

Computational Details

Theoretical methods

For the calculations we have used the M06-2X/def2-QZVP level of theory[10] and the Gaussian-16 program.[11] The X-ray coordinates have been used for the calculations unless otherwise noted. We have used this procedure because we are interested in the analysis of the interactions as they stand in the solid state. However, for the dimer of compound **12** we have fully optimized the geometry without any restriction. The NBO,[12] QTAIM[13] and NCIplot calculations[14,15] have been computed using M06-2X/def2-QZVP level of theory. The AIM analysis has been performed using the AIMAll program.[16] The interaction energy of complex **7** and a model to estimate the π-stacking interaction have been computed M06-2X/def2-TZVP level of theory using the optimized geometries.

The NCIplot index[14] facilitates the visualization and identification of non-covalent interactions. This efficient[15] index is based on the peaks that appear in the reduced density gradient (RDG) at low densities. The formation of a supramolecular complex induces a significant change in the RDG at the critical points in between the interacting molecules due to the annihilation of the density gradient at these points. Consequently, the NCI plot is useful to evaluate host–guest complementarity and the extent to which noncovalent forces stabilize a complex. The information is basically qualitative showing which molecular regions interact using a color scheme (red-yellow-green-blue scale). Red and blue colors are used to represent repulsive (ρ^{+}_{cut}) and attractive (ρ^{-}_{cut}) interactions, respectively. The yellow and green colors are used to represent weak repulsive and weak attractive forces, respectively.

Optimized geometry of complex 12

Figure S32 shows the fully optimized geometry of the heterodimer observed in the solid-state structure of compound **12**. In spite of the expected electrostatic repulsion, the dimer is stable in the

gas phase and exhibits a short $I^{+}\cdots Ag^{+}$ distance (3.210) strongly supporting that this contact is not merely a consequence of the crystal packing. The coordination distances are in reasonable agreement with the experimental ones (I^{+}–N bond distance 2.24 Å; average Ag^{+}–N distance 2.45 Å). The angle between the two NC5 planes of the 4 DMAP aromatic rings is 30.75º, which is in excellent agreement with the experimental one (32.9º), giving reliability to the theoretical method. The binding energy of isolated complex **12** in the gas phase is repulsive (32.4 kcal/mol) due to the electrostatic cation···cation repulsion (the counter-ions are not included in the calculation).

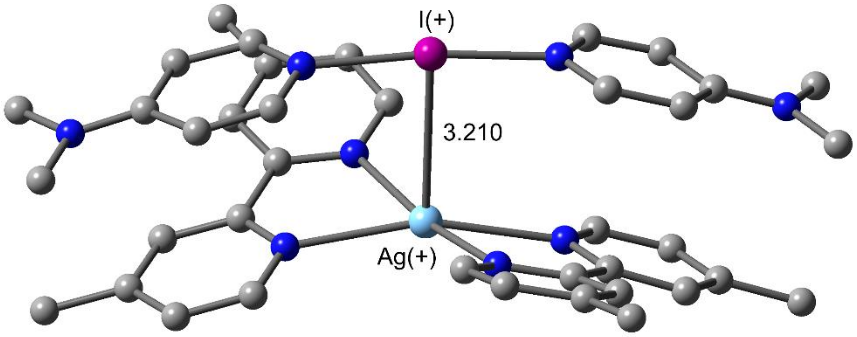

12

Figure S32: *M06-2X/def2-TZVP optimized geometry of **12** (distance in Å).*

Theoretical analysis of the contribution of π-stacking interactions in 12

Figure S33 shows a theoretical model used to evaluate the π-stacking interaction in complex **7**. We have simply used a model where the iodonium atom has been eliminated and the rest of the complex has been kept frozen. The interaction energy is –17.0 kcal/mol, thus evidencing that each π-stacking contributes in approximately –8.5 kcal/mol. It should be mentioned that this is a rough estimation of the π-stacking since in the real system, the presence of the I^{+} coordinated to the 4-DMAP significantly polarizes the aromatic ring.

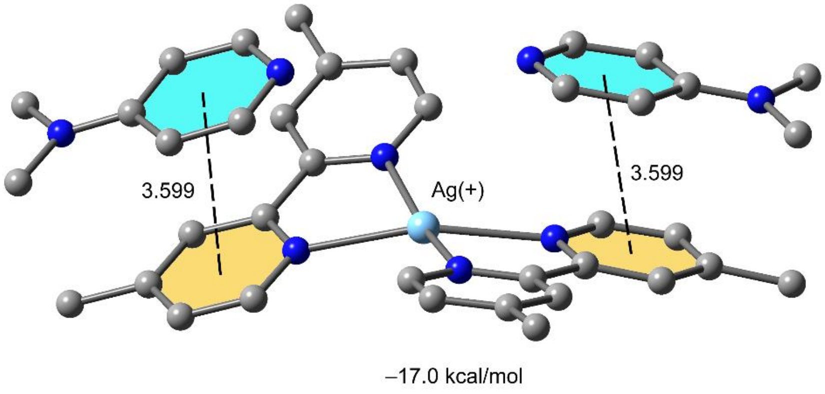

*Figure S33: Interaction energy of the theoretical model of complex **12** with indication of the π-stacking interaction.*

HOMO-LUMO plot of $[I(4\text{-DMAP})_2]^+$

Figure S34 shows the HOMO and LUMO plots of the optimized $[I\,(4\text{-DMAP})_2]^+$ fragment of compound **12**. The HOMO shows the participation of the p_z atomic orbital of the I-atom along with the π-systems of the 4-DMAP rings. This confirms the nucleophilic character of the I-atom in the $[I\,(4\text{-DMAP})_2]^+$ fragment, perpendicularly to the molecular plane (z-direction).

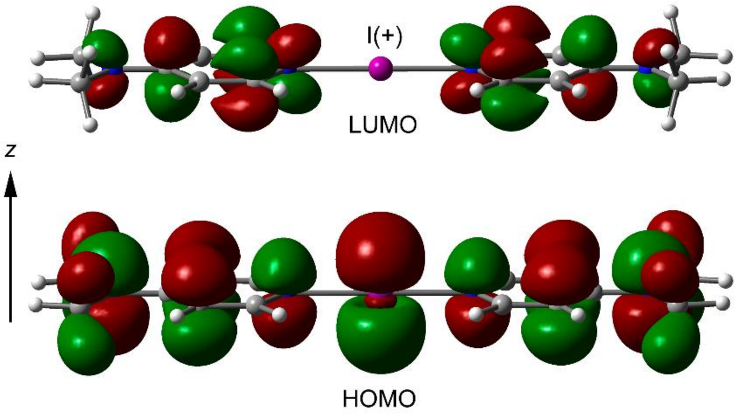

Figure S34: HOMO-LUMO plots of $[I\,(4\text{-DMAP})_2]^+$ using the 0.02 a.u. isosurface.

References

(1) Hooft, R. W. W.; Nonius. Collect. Nonius BV: Delft, The Netherlands 1998.

(2) Otwinowski, Z.; Minor, W. B. T.-M. in E. [20] Processing of X-Ray Diffraction Data Collected in Oscillation Mode. In *Macromolecular Crystallography Part A*; Academic Press, 1997; Vol. 276, pp 307–326. https://doi.org/https://doi.org/10.1016/S0076-6879(97)76066-X.

(3) Agilent Technologies Ltd. CrysAlis Pro. Agilent Technologies Ltd: Yarnton, Oxfordshire 2014.

(4) Sheldrick, G. M. SHELXT – Integrated Space-Group and Crystal-Structure Determination. *Acta Crystallogr. Sect. A Found. Adv.* **2015**, *71* (1), 3–8. https://doi.org/10.1107/S2053273314026370.

(5) Dolomanov, O. V.; Bourhis, L. J.; Gildea, R. J.; Howard, J. A. K.; Puschmann, H. OLEX2 : A Complete Structure Solution, Refinement and Analysis Program. *J. Appl. Crystallogr.* **2009**, *42* (2), 339–341. https://doi.org/10.1107/S0021889808042726.

(6) Sheldrick, G. M. Crystal Structure Refinement with SHELXL. *Acta Crystallogr. Sect. C, Struct. Chem.* **2015**, *71* (Pt 1), 3–8. https://doi.org/10.1107/S2053229614024218.

(7) Yu, S.; Kumar, P.; Ward, J. S.; Frontera, A.; Rissanen, K. A 'Nucleophilic' Iodine in a Halogen-Bonded Iodonium Complex Manifests an Unprecedented I+···Ag+ Interaction. *Chem* **2021**, 10.1016/j.chempr.2021.01.003. https://doi.org/10.1016/j.chempr.2021.01.003.

(8) Ward, J. S.; Fiorini, G.; Frontera, A.; Rissanen, K. Asymmetric [N–I–N]+ Halonium Complexes. *Chem. Commun.* **2020**, *56* (60), 8428–8431. https://doi.org/10.1039/D0CC02758H.

(9) Spek, A. L. PLATON SQUEEZE: A Tool for the Calculation of the Disordered Solvent Contribution to the Calculated Structure Factors. *Acta Crystallogr. Sect. C* **2015**, *71* (1), 9–

18. https://doi.org/10.1107/S2053229614024929.

(10) Zhao, Y.; Truhlar, D. G. The M06 Suite of Density Functionals for Main Group Thermochemistry, Thermochemical Kinetics, Noncovalent Interactions, Excited States, and Transition Elements: Two New Functionals and Systematic Testing of Four M06-Class Functionals and 12 Other Function. *Theor. Chem. Acc.* **2008**, *120* (1), 215–241. https://doi.org/10.1007/s00214-007-0310-x.

(11) Frisch, M. J.; Trucks, G. W.; Schlegel, H. B.; Scuseria, G. E.; Robb, M. a.; Cheeseman, J. R.; Scalmani, G.; Barone, V.; Petersson, G. a.; Nakatsuji, H.; Li, X.; Caricato, M.; Marenich, a. V.; Bloino, J.; Janesko, B. G.; Gomperts, R.; Mennucci, B.; Hratchian, H. P.; Ortiz, J. V.; Izmaylov, a. F.; Sonnenberg, J. L.; Williams; Ding, F.; Lipparini, F.; Egidi, F.; Goings, J.; Peng, B.; Petrone, A.; Henderson, T.; Ranasinghe, D.; Zakrzewski, V. G.; Gao, J.; Rega, N.; Zheng, G.; Liang, W.; Hada, M.; Ehara, M.; Toyota, K.; Fukuda, R.; Hasegawa, J.; Ishida, M.; Nakajima, T.; Honda, Y.; Kitao, O.; Nakai, H.; Vreven, T.; Throssell, K.; Montgomery Jr., J. a.; Peralta, J. E.; Ogliaro, F.; Bearpark, M. J.; Heyd, J. J.; Brothers, E. N.; Kudin, K. N.; Staroverov, V. N.; Keith, T. a.; Kobayashi, R.; Normand, J.; Raghavachari, K.; Rendell, a. P.; Burant, J. C.; Iyengar, S. S.; Tomasi, J.; Cossi, M.; Millam, J. M.; Klene, M.; Adamo, C.; Cammi, R.; Ochterski, J. W.; Martin, R. L.; Morokuma, K.; Farkas, O.; Foresman, J. B.; Fox, D. J. Gaussian 16, Revision A.01. 2016, p Gaussian 16, Revision A.01, Gaussian, Inc., Wallin.

(12) Glendening, E. D.; Landis, C. R.; Weinhold, F. Natural Bond Orbital Methods. *WIREs Comput. Mol. Sci.* **2012**, *2* (1), 1–42. https://doi.org/10.1002/wcms.51.

(13) Bader, R. F. W. A Quantum Theory of Molecular Structure and Its Applications. *Chem. Rev.* **1991**, *91* (5), 893–928. https://doi.org/10.1021/cr00005a013.

(14) Contreras-García, J.; Johnson, E. R.; Keinan, S.; Chaudret, R.; Piquemal, J.-P.; Beratan, D. N.; Yang, W. NCIPLOT: A Program for Plotting Noncovalent Interaction Regions. *J. Chem. Theory Comput.* **2011**, *7* (3), 625–632. https://doi.org/10.1021/ct100641a.

(15) Johnson, E. R.; Keinan, S.; Mori-Sánchez, P.; Contreras-García, J.; Cohen, A. J.; Yang, W. Revealing Noncovalent Interactions. *J. Am. Chem. Soc.* **2010**, *132* (18), 6498–6506. https://doi.org/10.1021/ja100936w.

(16) Keith, T. A. AIMALL. TK Gristmill Software: Overland Park KS, USA 2013.
